# Ion trap and release dynamics enables nonintrusive tactile augmentation in monolithic sensory neuron

**DOI:** 10.1126/sciadv.adi3827

**Published:** 2023-10-18

**Authors:** Hyukmin Kweon, Joo Sung Kim, Seongchan Kim, Haisu Kang, Dong Jun Kim, Hanbin Choi, Dong Gue Roe, Young Jin Choi, Seung Geol Lee, Jeong Ho Cho, Do Hwan Kim

**Affiliations:** ^1^ Department of Chemical Engineering, Hanyang University, Seoul 04763, Republic of Korea.; ^2^ Department of Engineering Science and Mechanics, Pennsylvania State University, University Park, PA 16802, USA.; ^3^ School of Chemical Engineering, Pusan National University, Busan 46241, Republic of Korea.; ^4^ School of Electrical and Electronic Engineering, Yonsei University, Seoul 03722, Republic of Korea.; ^5^ Department of Chemical and Biomolecular Engineering, Yonsei University, Seoul 03722, Republic of Korea.; ^6^ Department of Organic Material Science and Engineering, Pusan National University, Busan 46241, Republic of Korea.; ^7^ Institute of Nano Science and Technology, Hanyang University, Seoul 04763, Republic of Korea.; ^8^ Clean-Energy Research Institute, Hanyang University, Seoul 04763, Republic of Korea.

## Abstract

An iontronic-based artificial tactile nerve is a promising technology for emulating the tactile recognition and learning of human skin with low power consumption. However, its weak tactile memory and complex integration structure remain challenging. We present an ion trap and release dynamics (iTRD)–driven, neuro-inspired monolithic artificial tactile neuron (NeuroMAT) that can achieve tactile perception and memory consolidation in a single device. Through the tactile-driven release of ions initially trapped within iTRD-iongel, NeuroMAT only generates nonintrusive synaptic memory signals when mechanical stress is applied under voltage stimulation. The induced tactile memory is augmented by auxiliary voltage pulses independent of tactile sensing signals. We integrate NeuroMAT with an anthropomorphic robotic hand system to imitate memory-based human motion; the robust tactile memory of NeuroMAT enables the hand to consistently perform reliable gripping motion.

## Teaser

Ion trap and release dynamics–driven monolithic artificial tactile neuron can simultaneously perceive and learn tactility.

## INTRODUCTION

**Fig. 1.**
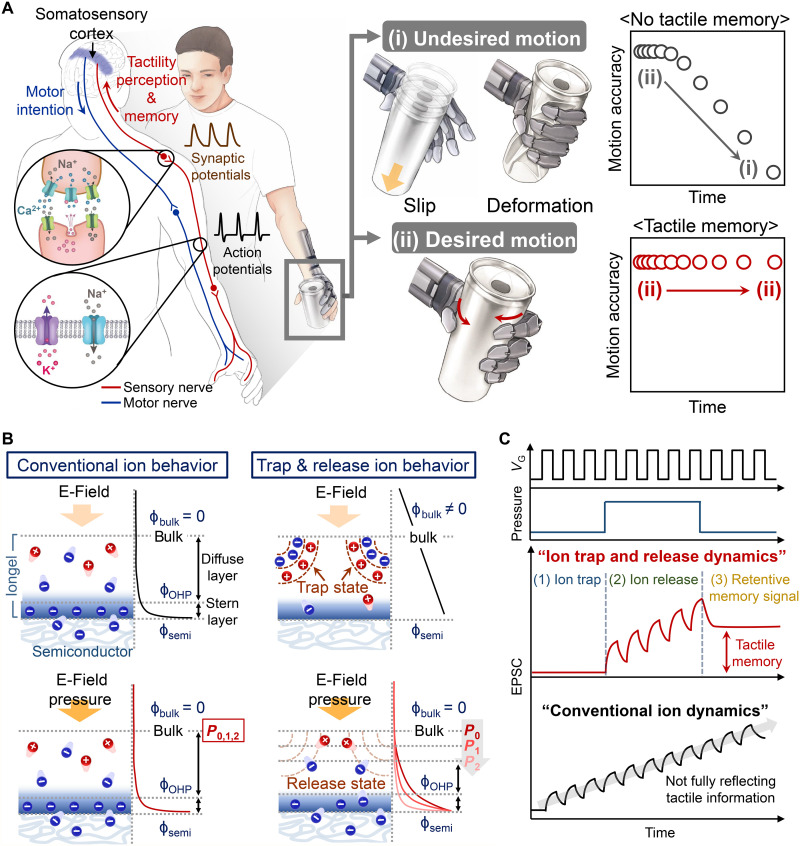
Concepts of tactile memory–based human motion and ion trap and release dynamics (iTRD)-driven neuro-inspired monolithic artificial tactile neuron (NeuroMAT). (**A**) Schematics of tactile perception and learning procedure in biological tactile neuron and tactile memory–based reliable human motion. (**B**) Comparison of conventional ion behavior and iTRD mechanism under external E-field and tactile stimuli. (**C**) Tactile perception and learning characteristics of iTRD-driven NeuroMAT.

Tactile memory is a crucial sensory modality that enables human skin to perceive and interpret its surrounding environment—vital tasks in human daily activities (*1*–*3*). The tactile encoding enables humans to identify objects via touch and reliably and repeatedly handle (e.g., grip and lift) them nondestructively through automatic access to previously acquired tactile information (i.e., motor skills) (*3*, *4*). This ability originates from tactile-based implicit memory [unconscious long-term memory (LTM)], enabling humans to rapidly and efficiently perform specific motion tasks without consciously retrieving object information (*1*, *5*). Such biological tactile recognition and LTM-based motion processing, realized by the tactile neural network of the somatosensory system, are essential functions that a neuro-inspired robotic system performing human motion must emulate. These functions can facilitate a human-like (anthropomorphic) robot to rapidly interact with a dynamic environment and consistently manipulate target objects without reduction of motion accuracy and requirement of iterative control command (Fig. 1A) (*3*, *4*, *6*, *7*).

Several artificial tactile neural systems, e.g., triboelectric- or ferroelectric-driven single-synaptic devices, have been proposed for mimicking biological tactile perceptual memory performance (*8*–*10*). The systems showed plausible LTM toward tactile stimuli but have the complex tactile perception (magnitude, frequency, and duration) or high-voltage operation, which are undesirable characteristics in artificial tactile neuromorphic devices (*11*). On the other hand, neural processing of sensory information in biological synapses, driven by charges (particularly ions), has inspired artificial synapse disciplines to exploit extremely low power consumption and high data processing efficiency (*11*–*14*). Approaches based on this concept primarily involve the integration of tactile sensors (triboelectric, piezocapacitive, or piezoresistive), signal converters (ring oscillator or ionic cable), and iongel-gated synaptic memory transistors (*15*–*18*) or fabrication of a suspended iongel-gated synaptic transistor with an additional control gate electrode (*19*). However, they suffer from complicated electrical interconnections, low circuit compatibility, data latency, and additional power requirements for operating each device component (*19*, *20*). The major drawback is a lack of tactility-induced LTM (typically <200 s), which is a critical feature for incorporating learning behavior in an artificial tactile neural system (table S1). This results from the intrinsic behavior of ions to reach an electrochemical equilibrium state within conventional iongel dielectrics in synaptic transistors. Ions accumulated on or diffused into the semiconductor layer can easily dissipate at zero voltage, which causes rapid erasure of the conductance encoded by the synaptic device (i.e., volatile tactile memory).

In the case of the integrated tactile neural system, voltage inputs transmitted from tactile sensors to synaptic transistors are tactile event–driven, thereby causing difficulty in the independent application of auxiliary voltage stimuli to suppress the ion equilibrium behavior for preserving the induced tactile memory. Even if external voltage stress is intentionally applied, the histories of the tactile event–driven synaptic signal and memory are easily distorted because the internal ion flux (i.e., ion migration and diffusion) in the iongel dielectrics is dominantly manipulated by voltage signals not generated by the tactile signals of the system. From this perspective, the ion behavior of conventional ionic materials poses a challenge to the augmentation of tactile memory and the design of a simple artificial tactile neural system capable of perceiving and learning tactile stimuli in a monolithic device.

Here, we report a neuro-inspired, monolithic (capable of sensing, memorizing, and even learning, all in a single device) artificial tactile neuron, termed NeuroMAT, with nonintrusive and augmented memory based on ion trap and release dynamics (iTRD), in which the free ion density and ion flux can be independently modulated using tactile stimuli (pressure) and an external electric field (E-field), respectively. We use this system to demonstrate a reliable human motion–mimicking an anthropomorphic robotic hand. In the ion trap state (*n*_free ions_ ≈ 0), the ion flux of the iTRD-iongel is not modulated by the external E-field; hence, the potential drop is uniformly distributed over the iongel film (Fig. 1B). This behavior is the opposite of that in a conventional iongel, wherein the potential drop is concentrated at the iongel–semiconductor layer interface because of E-field–induced formation of an electric double layer (EDL). Tactile stimulus (pressure) application can release the trapped ions (*n*_free ions_ ≠ 0), at which instant only the released ions can contribute to the total ion flux of the iTRD-iongel; this consequently causes a considerable potential drop in the interface regions through released ion-induced EDL formation. This implies that the ions released under tactile stimuli can exclusively generate synaptic memory signals (reflecting tactile information), and the induced memory level can be augmented by applying an auxiliary external E-field (suppression of equilibrium behavior of the released ions). We used this unique ion behavior to develop the iTRD-driven NeuroMAT with nonintrusive and augmented memory, capable of simultaneously sensing, memorizing, and learning tactile stimulation (Fig. 1C). The NeuroMAT can perform highly sensitive tactile recognition under a series of voltage pulses; concurrently, retention of the encoded tactile memory is effectively improved by the application of voltage signals independent of the tactile signals. This characteristic cannot be explored in conventional iongel-based synaptic transistors because the internal ion flux is already dominated by the E-field so that ion-induced synaptic signal cannot be manipulated by tactile stimuli.

## RESULTS

### A concept proof of iTRD

**Fig. 2.**
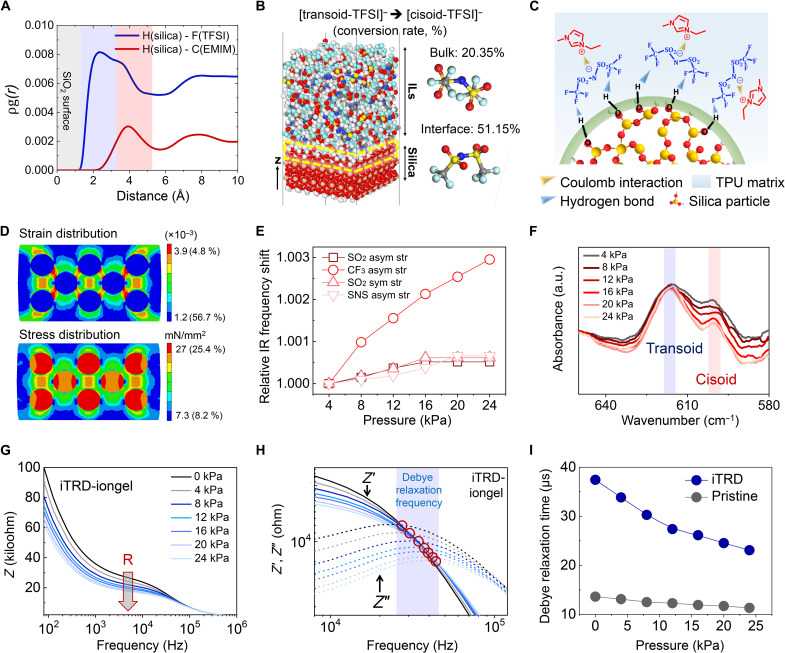
Molecular design and ion dynamics of iTRD-iongel for NeuroMAT. (**A**) Pair correlation function of hydrogen (silica)–fluorine (TFSI) (blue line) and hydrogen (silica)–carbon of side chain (EMIM) (red line). (**B**) Equilibrated structures of the bulk model of [EMIM]^+^[TFSI]^−^ and silica slab model with the conformational conversion rate. (The white, gray, blue, red, cyan, yellow, and orange symbols correspond to H, C, N, O, F, S, and Si atoms, respectively.) (**C**) Schematic of demonstrated molecular structure and ion configuration of iTRD-iongel. (**D**) Finite element method simulation showing effective stress and strain of iTRD-iongel under applied pressure. (**E**) Relative changes in infrared (IR) absorption frequency (peak position) in spectral ranges of 1330 to 1380 cm^−1^, 1200 to 1250 cm^−1^, 1120 to 1150 cm^−1^, and 1040 to 1070 cm^−1^, which correspond to SO_2_ asymmetric, CF_3_ asymmetric, SO_2_ symmetric, and SNS asymmetric stretching (asym str) vibration bands, respectively. (**F**) Fourier transform infrared (FTIR) spectra corresponding to transoid and cisoid configurations of [TFSI]^−^ in the 580- to 670-cm^−1^ range. (**G**) Frequency-dependent complex impedance spectra of iTRD-iongel under various pressures (0 to 24 kPa). (**H**) Frequency-dependent real (solid line) and imaginary (dashed line) impedance spectra of iTRD-iongel under various pressures (0 to 24 kPa). (**I**) Comparison of variation tendencies of Debye relaxation times of pristine iongel and iTRD-iongel as a function of applied pressure. a.u., arbitrary units.

To systematically prove the concept of iTRD, the ion trap state of the iTRD-iongel, wherein silica microparticles coordinated with ionic liquids {ILs; 1-ethyl-3-methylimidazolium bis(trifluoromethyl sulfonyl)imide ([EMIM]^+^[TFSI]^−^)} are dispersed in the thermoplastic polyurethane (TPU) polymer matrix, should be investigated. To this end, we performed molecular dynamics (MD) simulations to explore the molecular configurations of the ILs on the surface of the silica microparticles used as ion trap sites. Since [EMIM]^+^ and [TFSI]^−^ are interacted by ionic Coulombic interactions, it needs to clarify which side of the IL was trapped on the silica microparticle surface. To understand the structural relationship between [EMIM]^+^[TFSI]^−^ and the silica microparticle surface, we analyzed the pair correlation function (PCF) of hydrogen (silanol of silica)–fluorine ([TFSI]^−^) and hydrogen (silanol of silica)–carbon of the side chain (−CH_2_−CH_3_ in [EMIM]^+^) (Fig. 2A). The intensities of the fluorine and carbon components were higher than those of the other pairs, indicating that these two components were the main constituents of [TFSI]^−^ and [EMIM]^+^, respectively, on the silica microparticle surface (fig. S1). The PCF curve of hydrogen (silanol of silica)–fluorine ([TFSI]^−^) began at a shorter distance (1.2 Å) from the silica surface (hydrogen atom) than the carbon of the side chain ([EMIM]^+^) (2.3 Å). Furthermore, the calculated coordination number of hydrogen (silanol of silica)–fluorine ([TFSI]^−^) was 4.91, which is almost 3.3 times that (1.49) of hydrogen (silica)–carbon of the side chain ([EMIM]^+^). These results indicate that [TFSI]^−^ was densely concentrated on the silica surface, and the molecular interaction between the fluorine atoms and the silanol groups provided a driving force for the ion trap state. This intermolecular interaction of [TFSI]^−^ can influence its molecular conformation state. From the slab model of the canonical ensemble MD simulation, the configuration conversion rate from [transoid-TFSI] to [cisoid-TFSI] on the silica surface was established as 51.15 ± 3.31%, which is higher than that in the bulk phase of the ILs (20.35 ± 8.51%) (Fig. 2B, fig. S2, and movie S1). This configurational alteration is attributed to the hydrogen bonds (HBs) between the silanol groups of the silica microparticles and [TFSI]^−^ (fig. S3) (*21*, *22*), and the HBs serve as a building block for achieving the ion trap state (*22*, *23*). In addition, as a result of the ion trap state, the initial capacitance of the iTRD-iongel exhibited a considerable reduction compared to that of the pristine iongel, although both films had an identical loading amount of the ILs (20 wt % by the weight of TPU matrix) (fig. S4). Consequently, the [cisoid-TFSI] could be trapped in the silanol groups of the silica microparticle surface by the HBs, which is the proposed ion trap state of the iTRD-iongel (Fig. 2C).

The ion release states of the ILs trapped on the silica microparticle surface can be activated by relaxing the HBs using external mechanical stimuli. A finite element method (FEM; tactile) simulation showed that, under pressure, the mechanical stress and strain induced within iTRD-iongel were concentrated mainly at the silica microparticle–TPU matrix interface because of an elastic modulus (*E*) mismatch (30 GPa and 10 MPa, respectively) (Fig. 2D). Unlike the iTRD-iongel, the stress and strain within the pristine iongel were broadly distributed over the entire film (fig. S5). The localized mechanical energy at the interfaces can be dissipated by the HBs between the silanol groups and [TFSI]^−^ (*24*). The mechanical stress focused on the ion-trap region can increase the distance between [TFSI]^−^ and the silica surface, thereby effectively reducing the binding energy of the HBs (fig. S6). Last, these HBs can be cleaved at a pressure sufficiently high to separate the ions from the silica microparticles. To validate the mechanosensitive ion release mechanism, we investigated the molecular configuration change of [TFSI]^−^ through Fourier transform infrared (FTIR) analysis under tactile stimulation. From the above-presented discussion of the MD simulation, it can be speculated that as the trapped ions are released, the ion configuration would be modified from [cisoid-TFSI] to [transoid-TFSI], resulting in changing the nature of its molecular vibration. Under pressure, the vibrational band of [TFSI]^−^—ranging from 1000 to 1400 cm^−1^—shifted toward a higher wave number, indicating an improvement in the degree of freedom of molecular motion of [TFSI]^−^ (fig. S7) (*25*). The shift in the CF_3_ asymmetric stretching peak corresponding to the main molecular component tethered to the silanol groups was particularly prominent (Fig. 2E). This means that the [TFSI]^−^ trap state could be altered under mechanical stimuli, causing a change in its molecular configuration. With increasing intensity of the pressure applied to the iTRD-iongel, the infrared peak intensity of [cisoid-TFSI] decreased relative to that of [transoid-TFSI], implying a decrease in the number of [cisoid-TFSI] structures on the silica surface (Fig. 2F). These results are key evidence for the release of ions trapped on the silica microparticle surface with a change in their molecular configuration via tactile stimulation, which also agrees with the MD simulation results.

The free ion concentration (*c_i_*) in the iTRD-iongel is expected to increase with the release of trapped ions under mechanical stimuli, which would influence the ion dynamics associated with *c_i_*, e.g., ionic conductivity ($\sigma ={q_{i}}^{2}c_{i}D_{i}/kT$, where *q_i_*, *D_i_*, and *kT* are the ion charge, ion diffusivity, and temperature in the unit of energy, respectively) and Debye screening length ($\kappa^{-1}=\sqrt{\varepsilon_{r}\varepsilon_{0}kT/{q_{i}}^{2}c_{i}}$, where ɛ*_r_* and ɛ_0_ are the dielectric constant and space-charge permittivity, respectively) (see Supplementary Text) (*26*). In the Bode plot of the pristine iongel, the complex impedance (*Z* = *l*/σ*A* + *R_e_*, where *l*, *A*, and *R_e_* are the thickness, contact area, and electrode resistance, respectively) in the intermediate-frequency region (approximately 10^3^ to 10^5^ Hz) remained unchanged under tactile stimuli (fig. S8A). Meanwhile, the impedance of the iTRD-iongel gradually decreased with increasing pressure intensity, indicating that the ionic conductivity could be improved by tactile stimulation (Fig. 2G and fig. S9). To reveal that the origin of the enhanced ionic conductivity was attributed to the *c_i_* increase in the iTRD-iongel, a variation in the Debye screening length (κ^−1^) with applied tactile stimuli was investigated (see Supplementary Text). In frequency-dependent real part (*Z*′) and imaginary part (*Z*″) impedance spectra, the frequency at the intersection position of *Z*′ and *Z*″ indicates Debye relaxation frequency ($\omega_{D}={\tau_{D}}^{-1}$, where τ*_D_* is Debye time given by 1/κ^2^*D*) (*27*); in the Bode plot of the iTRD-iongel, the defined Debye relaxation frequency noticeably shifted toward higher frequencies with increasing pressure, which was not observed tendency in the plot of the pristine iongel (Fig. 2H and fig. S8B). Compared to the pristine iongel, the Debye relaxation time of the iTRD-iongel gradually decreased as applied pressure increased (Fig. 2I), implying that the ions released by the tactile stimulation were responsible for the increased *c_i_* (shortened κ^−1^) in the iTRD-iongel. Note that the shift tendency of the Debye relaxation frequency with applied pressure in iTRD-iongel was analogous to that in the pristine iongel under increasing loading amounts of ILs (fig. S10).

### Characterization of iTRD-driven NeuroMAT

**Fig. 3.**
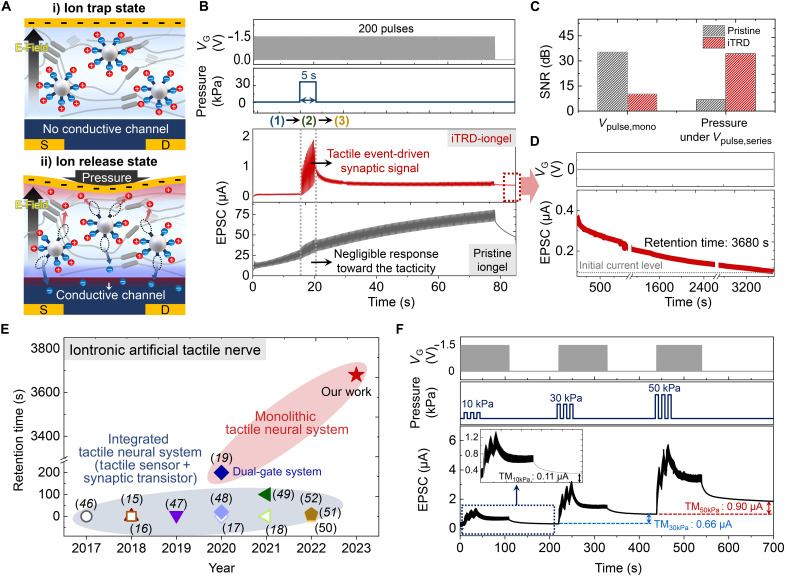
Tactile perception and learning behavior of NeuroMAT. (**A**) Schematics of the ion trap and release state in NeuroMAT, depicting the transition of ion behavior depending on applied tactile stimulation. (**B**) Comparison of tactile perception performances of pristine iongel-based synaptic transistors and NeuroMAT under application of series of *V*_G_ pulses. (**C**) Signal-to-noise ratio (SNR) characteristics of pristine iongel-based synaptic transistors and NeuroMAT under single *V*_G_ pulse and tactile stimulation along with series of *V*_G_ pulses. The collection of baseline data for the SNR calculation is shown in fig. S24. (**D**) Retention time of the induced tactile memory (long-term memory) of NeuroMAT. (**E**) Comparisons of the previously demonstrated tactile memory of iontronic artificial tactile nerves with our work. (**F**) Tactile detection and memory characteristics of NeuroMAT under dynamic tactile stimulation (10, 30, and 50 kPa).

Because of the mechano-triggered ion trap and release behavior, the iTRD-iongel showed a larger capacitance change against tactile stimuli than the pristine iongel (fig. S11). Furthermore, the capacitive variation in response to repeated tactile stimuli continuously exhibited reliable output signals, proving the high mechanical durability of iTRD-iongel (fig. S12). Note that the pressure sensitivity demonstrated by iTRD-iongel was found to be higher than that of a homogeneously designed iongel system with ion confinement (*28*). This observation suggests that the heterogeneous molecular design of iTRD-iongel, which incorporates silica microparticles within the TPU matrix, enables a more effective tactile perception by harnessing the concentrated mechanical force at the interface, as discussed earlier in Fig. 2D and fig. S5. On the basis of this, NeuroMAT, which is an iTRD-iongel–gated synaptic transistor based on *p*-type polymer semiconductor {poly[2,5-bis(2-decyltetradecyl)-3-{5-(thieno[3,2-b]thiophen-2-yl)thiophen-2-yl}-6-(thiophen-2-yl)pyrrolo[3,4-c]pyrrole-1,4(2H,5H)-dione (PDPPTT)]}, can achieve pressure-sensitive current modulation (Fig. 3A and figs. S13 and S14). Therefore, NeuroMAT could control its activity-dependent synaptic plasticity (e.g., paired-pulse facilitation) depending on applied tactile information, which was not available in the pristine iongel–based synaptic transistors (figs. S15 and S16). This is because, in the iTRD-iongel, only released ions by tactile stimuli could contribute to conductive-channel formation in the polymer semiconductor layers. Under a series of gate voltage (*V*_G_) pulses without pressure, a conductive channel was hardly formed in NeuroMAT because of the ion trap state in the iTRD-iongel; however, when mechanical stimulus is applied along with the negative *V*_G_ stimulation, the released [TFSI]^−^ ions can penetrate into the semiconductor layer and consequent formation of a high-conductance channel (Fig. 3A). Since ion penetration into the semiconductor layer plays a key role in inducing tactile-driven LTM characteristics, the *V*_G_ pulse condition that would facilitate the infiltrating behavior of ions should be determined (*29*–*31*). *V*_G_ magnitude is one of the driving forces for ion injection into a channel layer. Ion penetration into the layer causes electrochemical doping in the entire bulk region. Consequently, the entire layer can act as a charge transport pathway; then, the electrical current of the channel is dependent on the film thickness (*32*). When −1.5-V *V*_G_ was applied to NeuroMAT, the electrical current was enhanced as the thickness of the PDPPTT layer was thicker (fig. S17). This indicates that the voltage magnitude is a critical factor for facilitating ion penetration, which is in good agreement with the observation of the polaron (i.e., doped) state in the semiconductor at −1.5-V *V*_G_ (fig. S18) (*33*, *34*). It was also confirmed that the capacitor with PDPPTT/iTRD-iongel showed resistive behavior (0° < θ < 45°) in combination with an increase of the capacitance at −1.5 V in low-frequency regimes (<100 Hz), implying that ion penetration into the channel layer effectively occurred (fig. S19) (*32*). Along with the *V*_G_ magnitude, the number of *V*_G_ pulses should be considered to achieve deep ion penetration into the channel layer, which would delay back diffusion of the penetrated ions to the iongel. The bi-exponential decay curve model of NeuroMAT (*31*) revealed a delay of relaxation of the doped states (τ_de − doping_) with an increasing number of *V*_G_ pulses (figs. S20 and S21). Consequently, the tactile perception and memory capabilities of NeuroMAT were investigated under the optimized *V*_G_ conditions (−1.5 V with a pulse width of 100 ms and more than 50 pulses).

As aforementioned discussion in Fig. 1, the pristine iongel–based (i.e., driven by conventional ion dynamics) synaptic transistor showed an inferior tactile response under auxiliary *V*_G_ pulses (Fig. 3B). This is strong evidence for the unsuitability of conventional ion dynamics to realize a monolithic artificial tactile neuron platform. In contrast, when a tactile stimulus was absent, NeuroMAT did not respond under the application of the series of *V*_G_ pulses; however, clear synaptic signals were generated when pressure was applied along with the *V*_G_ (Fig. 3, A and B). The tactile recognition performance of NeuroMAT was well-defined even under dynamic mechanical stimuli (fig. S22) because of the mechanoselective signal-to-noise ratio (SNR) property of the iTRD. Compared to the pristine iongel-based device, NeuroMAT showed negligible excitatory postsynaptic current signals under a single *V*_G_ pulse because of the ion trap behavior (fig. S23). When mechanical stimulation was applied under successive *V*_G_ pulses, the SNR property of NeuroMAT was completely reversed by the released ions [34.3 dB; this value was 5.1 times higher than that of the pristine iongel–based synaptic transistor (6.7 dB)] (Fig. 3C), which enabled NeuroMAT to simultaneously perceive and learn tactile information in a monolithic device. As shown in Fig. 3D, the tactile-induced synaptic signals of NeuroMAT were successfully consolidated by auxiliary *V*_G_ pulses, so that the LTM characteristics were effectively developed (over 3600 s of retention time). Note that the tactile memory performance of NeuroMAT was even higher than the previously demonstrated iontronic artificial tactile nerves (Fig. 3E). Because of the continuous *V*_G_ stimulus, the energy consumption of NeuroMAT was not at an extremely low level for recognizing and learning tactile information (fig. S25). However, it remains comparable to that of conventional synaptic devices (*11*) and even lower than that of sensor-integrated tactile neural systems (*15*–*17*). This is remarkable considering the capability of NeuroMAT to perceive and memorize tactile stimuli simultaneously within its monolithic device structure. Furthermore, the higher the applied pressure, the higher the memory level induced (Fig. 3F). This is attributed to the increase in the stress distribution and magnitude near the silica microparticles as the applied pressure increased (fig. S26). This facilitates the relaxation of HBs and leads to the release of a greater number of the trapped ions within iTRD-iongel. The programming and erasing memory cycles of the induced tactile memory were also reliable enough for the reuse of NeuroMAT, indicating its considerable potential for practical application (fig. S27).

### Realization of tactile memory–driven robotic system

**Fig. 4.**
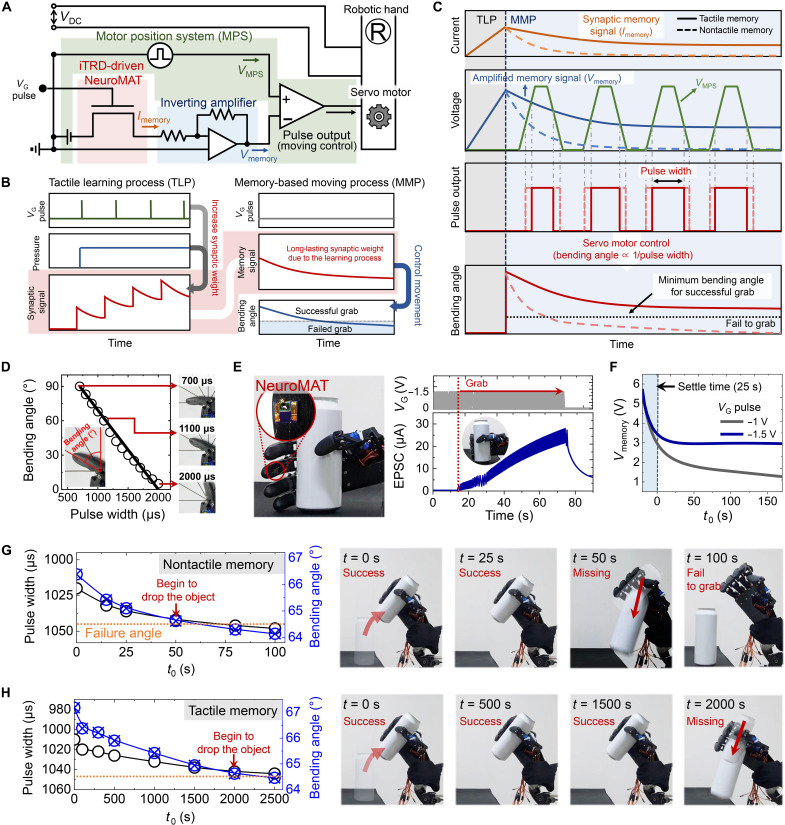
Design and demonstration of NeuroMAT-integrated neural circuit system (NeuroMATICS) for emulating tactile memory–based human motion. (**A**) Circuit diagram of tactile memory–based NeuroMATICS with NeuroMAT, inverting amplifier, motor position system, and anthropomorphic robotic hand. (**B**) Schematic diagram of signal transmission processes of tactile learning process (TLP) and memory-based movement process (MMP). (**C**) Detailed schematic diagram of MMP. (**D**) Plot of change in bending angle with change in width of pulse output. (**E**) Programming process of pressure required by NeuroMAT-integrated robotic hand to grip target object. (**F**) Comparison of *V*_memory_ values derived from nontactile and developed tactile memory states. (**G** and **H**) Variations in pulse width and bending angles depicted in photographic images showing the repeated gripping motion of the robotic hand in nontactile and tactile memory states, respectively, of NeuroMATICS.

To use the demonstrated tactile memory in human motion-inspired robotics, we fabricated a NeuroMAT-integrated neural circuit system (NeuroMATICS) by incorporating NeuroMAT into an anthropomorphic robotic hand. To operate the voltage-driven robotic hand using a synaptic memory current (*I*_memory_) from the NeuroMAT, it is necessary to amplify and convert *I*_memory_ into *V*_memory_. Figure 4A depicts the circuit diagram of NeuroMATICS, which comprises an inverting amplifier and a comparator. The operating mechanism of NeuroMATICS involves two processes: a tactile learning process (TLP) and a memory-based movement process (MMP) (Fig. 4B). In the TLP, *V*_G_ pulses and a specific pressure were applied to NeuroMAT, which resulted in the learning of tactile information (fig. S28). After the TLP, the generated tactile memory signal was transmitted to a servomotor to operate the anthropomorphic robotic hand for MMP, which is illustrated in detail in Fig. 4C. The *I*_memory_ from NeuroMAT was applied to an inverting amplifier to convert the *I*_memory_ into an amplified voltage signal (*V*_memory_). This *V*_memory_ signal was then transmitted to the comparator in a motor position system (MPS), which is responsible for controlling the positioning of the servomotor in the robotic hand (Fig. 4A). In the comparator, *V*_memory_ was compared to a trapezoidal-shaped waveform referred to as *V*_MPS_. When *V*_MPS_ exceeded *V*_memory_, pulse output signals were sent to the servomotor of the robotic hand (Fig. 4C). The width of the pulse output was determined by the width of the intersection points between the trapezoidal-shaped *V*_MPS_ and *V*_memory_. Note that the pulse width regulated the bending angle of the robotic hand, where a larger pulse width resulted in a smaller bending angle (Fig. 4D and fig. S29). This implies that a rapid decay of *V*_memory_ led to wider pulse output, causing incomplete gripping motion of the robotic hand over time.

To mimic tactile memory–induced human motion using NeuroMATICS, NeuroMAT was attached to the robotic hand and programmed the required force by the TLP when the robotic hand properly gripped the proposed object (Fig. 4E). Application of −1.5-V *V*_G_ pulses to NeuroMATICS prolonged the induced tactile memory (fig. S30); however, in the case of −1 V *V*_G_ pulses, tactile memory characteristics did not develop effectively (fig. S31), which agrees with the discussion of Fig. 3. Consequently, *V*_memory_ showed slow decay behavior with successful development of the tactile memory characteristics of NeuroMAT (Fig. 4F), indicating that long retention of the tactile memory would enable the robotic hand to consistently manipulate the object with high reliability. *V*_memory_ for NeuroMATICS with nontactile memory (*V*_G_ pulse: −1 V) decreased markedly for 100 s, and the pulse width and bending angle consequently changed from 1020 to 1048 μs and from 66.38° to 64.14°, respectively (Fig. 4G and fig. S32A). Because the critical bending angle for successful gripping and lifting is 64.5°, after 50 s, the robotic hand began missing the object while attempting to lift it; after 80 s, it stopped operating. However, when NeuroMATICS had tactile memory (*V*_G_ pulse: −1.5 V), the robotic hand could consistently and reliably perform gripping and lifting motions over 2000 s (pulse width: 1042 μs, bending angle: 64.62°) (Fig. 4H, fig. S32B, and movie S2). This result implies that the development of robust tactile memory in human-inspired robotics enabled successful and reliable emulation of human motion.

## DISCUSSION

In summary, we developed an iTRD-driven, neuro-inspired monolithic artificial tactile neuron (NeuroMAT) with nonintrusive and augmented memory. The ion behavior of the iTRD is completely distinguished from conventional ion dynamics in that the free ion density and ion flux can be independently modulated by tactile and voltage stimulations, respectively. The mechano-driven iTRD mechanism enabled the proposed NeuroMAT to simultaneously perceive, memorize, and learn tactile information in a single device, without requiring any additional sensors and memory devices. We used the augmented tactile memory of NeuroMAT to develop a NeuroMATICS-based robotic hand system, which consistently and reliably emulated tactile memory–driven human motion. Our theoretical study and practical demonstration of the iTRD behavior can provide inspiration for artificial nerve disciplines and can be extended to the field of neuromorphic electronic skin and machine learning.

## MATERIALS AND METHODS

### Materials

Chlorobenzene (CB), *N*,*N*-dimethylformamide (DMF), acetone, isopropanol (IPA), and hydrochloric acid were purchased from Sigma-Aldrich and were used as received. Tetraethyl orthosilicate (TEOS) for the synthesis of silica microparticles was also purchased from Sigma-Aldrich. The polymer semiconductor, PDPPTT (molecular weight: 521 kDa; polydispersity index: 3.34), was purchased from Brilliant Matters. TPU beads (KA-480) were purchased from Kolon Industries Inc. ILs, [EMIM]^+^[TFSI]^−^, were purchased from Solvionic. Parylene-C (OBT-PC300) was provided by Obang Technology.

### Simulation methods

MD simulations were performed to investigate the molecular configurations of [EMIM]^+^[TFSI]^−^ on the silica microparticle surface. Model of the bulk complex of [EMIM]^+^[TFSI]^−^ was generated using a periodic boundary condition (PBC) with dimensions of 44.05 Å by 44.05 Å by 44.05 Å. The interactions of the [EMIM]^+^[TFSI]^−^ model in the MD simulations were described using a modified classical force field based on the all-atom optimized potentials for liquid simulations (OPLS-AA) (*35*)/assisted model building with energy refinement (AMBER) (*36*) framework. All MD simulations were performed using the large-scale atomic/molecular massively parallel simulator (*37*). Volume annealing procedures were applied to effectively obtain the equilibrated structure; these procedures comprised the following steps: (i) the temperature was increased from 0 to 298.15 K for 300 ps in a canonical (NVT) ensemble MD simulation; (ii) the volume of the bulk model was gradually expanded to 200% of the initial volume for 100 ps while gradually increasing the temperature from 298.15 to 600 K; (iii) the bulk model was retained for 100 ps at 600 K; (iv) the volume of the bulk model was then gradually reduced to its initial volume for 100 ps while decreasing the temperature from 600 to 298.15 K; (v) the bulk model was retained for 100 ps at 298.15 K. The volume annealing in steps (ii) to (v) was repeated three times. Consequently, the temperature annealing procedure was applied, which comprised steps (ii) to (v) of the volume annealing procedure but without volume control. The temperature annealing procedure was also repeated three times. Then, the annealed model was subjected to an isothermal–isobaric ensemble (NPT) MD simulation for 5 ns at 298.15 K and 1 atm. After NPT simulation for an additional 5 ns for data collection, the IL model density was calculated to be 1.523 g/cm^3^, which was in good agreement with the experimental density (1.52 g/cm^3^). Then, the silica slab model was generated using a PBC with dimensions of 39.82 Å by 39.82 Å by 300.00 Å. A large vacuum space was assigned in the *z*-axis direction to prevent unnecessary interactions beyond the PBC. A hydroxylated (0 0 1) surface of α-cristobalite was constructed for a silica microparticle surface with a concentration of 8 OH/nm^2^ (*38*). The slab model was equilibrated by the NVT MD simulation for 5 ns at 298.15 K and 1 atm. The velocity Verlet algorithm with a time step of 1 fs was used for the motion of each atom (*39*), and the particle-particle particle-mesh method was adopted to calculate the long-range correction for electrostatic interactions (*40*). Consequently, the NVT MD simulation was performed for an additional 5 ns for data collection.

Density functional theory (DFT) calculations were performed to estimate the binding energy curve as a function of the distance between silica microparticles and [TFSI]^−^. A silica model for the adsorption of [TFSI]^−^ molecule was generated using a PBC with dimensions of 14.93 Å by 9.96 Å by 40.00 Å. The Vienna Ab initio Simulation Package was used for all DFT calculations (*41*). The projector-augmented wave method was used with the generalized gradient approximation and the Perdew-Burke-Ernzerhof functional (*42*). The energy and force convergences were 1 × 10^−6^ eV and 0.01 eV A^−1^, respectively, with an energy cutoff of 500 eV. Brillouin zone integration was performed using the Monkhorst-Pack method at 2 × 3 × 1 k-points (*43*).

FEM simulations were performed using the commercially available FEM software ADINA to investigate the effective stress and strain distributions in the iTRD-iongel. The thickness of the iTRD-iongel and the diameter of the silica microparticles were modeled to be 200 and 50 μm, respectively. The Young’s moduli of the TPU matrix and silica microparticles were modeled to be 10 MPa and 70 GPa, respectively. An input pressure of up to 100 kPa was gradually applied using a rigid (nondeformable) plate above the iTRD-iongel. The bottom faces of the models were set as the boundary conditions.

### Fabrication and characterization of pristine iongel and iTRD-iongel

The iTRD-iongel was synthesized by an in situ biphasic sol-gel process. To this end, TEOS was added to distilled water in a weight ratio of 2:1, and the mixture was stirred at 40°C for 10 min. Then, 20 wt % of the ILs was added dropwise to the TEOS-water mixture and stirred for an additional 15 min at the same temperature. Subsequently, hydrochloric acid (0.06 M) was added dropwise to this mixture in a weight ratio of 1:15. When hydrochloric acid is added to a TEOS-water-IL mixture, hydrolysis and condensation reactions of TEOS begin immediately, which results in the formation of a silica network (Si-O-Si) in which Si-OH groups are dominantly present on the surface (*44*). In our study, the silica-IL mixture was obtained after additional stirring for 15 min at 40°C. Next, the TPU solution was prepared by dissolving TPU beads in DMF in a weight ratio of 1:5 and stirring the resultant solution at 80°C for 3 hours. Then, the prepared silica-IL mixture was added dropwise to the TPU solution under continuous stirring at 80°C, and the resultant silica-IL-TPU tricomponent mixture was stirred at 80°C for an additional 20 hours. Last, the iTRD-iongel film was formed by printing the silica-IL-TPU solution on Au-deposited substrate or polymer semiconductor–coated substrate by use of a dispenser and desktop robot (ML-808GX and SM200SX-3A, Musashi Engineering Inc.).

FTIR spectroscopy was performed using a custom-built pressure-dependent FTIR instrument for in situ observation of molecular interactions (e.g., the HBs between silanol groups and ILs) under mechanical stimulus. This FTIR instrument consisted mainly of a force gauge–integrated attenuated total reflection (ATR) accessory unit (Golden Gate, Specac), pressure control unit, and spectrometer unit (INVENIO-R, Bruker Optics GmbH). A contact region was formed between the solid film and the ATR crystal by altering the *z* axis of the latter (ZnSe). At the same time, the applied pressure was measured in real time. The scan range of 4000 to 450 cm^−1^ was used for recording each spectrum under certain pressure conditions, and the final spectrum was considered to be the average of 64 scans with a resolution of 2 cm^−1^.

Electrochemical impedance spectroscopy measurements were performed using an electrochemical analyzer (PGSTAT302N, Metrohm Autolab) in a frequency range of 0.1 Hz to 100 kHz with a 10 mV AC signal. Impedance spectra were also measured under certain pressure conditions by inserting the force gauge probe into the top hole of the test cell (KP-Solid Cell, Hohsen Corp.). All impedance spectra were fitted with the corresponding equivalent circuit models generated using the NOVA program (Metrohm Autolab). The ionic conductivity (σ) was calculated using the following equation; $\sigma ={R_{B}}^{-1}A^{-1}L$, where *R*_B_, *A*, and *L* are the resistance, area, and thickness of the film, respectively. Capacitance measurements were performed by sandwiching the iTRD-iongel film between two Au-deposited polyethylene terephthalate substrates in a capacitive device configuration and connecting to them to an LCR meter (E4980, Agilent).

### Fabrication and characterization of iTRD-driven NeuroMAT

A heavily *n*-doped Si/SiO_2_ wafer (SiO_2_ thickness: 3000 Å) was used as a substrate; it was cleaned with acetone and IPA by ultrasonication. Cr/Au (3/20 nm) source/drain electrodes of the iTRD-driven NeuroMAT were thermally evaporated onto the substrate through a patterned shadow mask under a pressure of 8.0 × 10^−6^ torr (VTR 5006, SNTEK). Then, a parylene-C film (50 nm) was formed on the substrate to passivate the source/drain electrodes, and the channel area was patterned via reactive ion etching (Femto Science) for deposition of the polymer semiconductor (PDPPTT) layer. PDPPTT solution (5 mg/ml in CB) was spin-coated onto the substrate at 1000 rpm for 60 s, which was then annealed at 180°C for 3 hours in an N_2_ atmosphere. Subsequently, the prepared iTRD-iongel solution was printed onto the deposited PDPPTT layer using the dispenser and desktop robot. After printing of the iTRD-iongel, NeuroMAT was annealed on a hot plate for 3 hours at 80°C in an ambient atmosphere. The electrical properties of the devices were measured using a probe station (MS-TECH) connected to a semiconductor parameter analyzer (Keithley 4200). The electrical output signals generated under the applied voltage pulse were measured using a pulse measurement unit (Keithley 4225-PMU). Pressure was applied and measured using a custom-built sensor probe station equipped with a programmable *z*-axis stage with a force gauge (Mark-10, resolution of 0.002 N). UV-vis-NIR absorption spectra (400 to 2400 nm) of the iTRD-iongel with PDPPTT were recorded using a UV-vis-NIR spectrometer (V-770, JASCO) with a resolution of 0.5 nm at a scan rate of 1000 nm/min.

### Design and operation of NeuroMATICS

The iTRD-driven NeuroMAT, an inverting amplifier, an MPS, and an anthropomorphic robotic hand (RB-You-05, RobotShop Inc.) with a servomotor were integrated to fabricate a grip force memory system. Comparators (UA741CP, Texas Instruments) were used for the inverting amplifier and MPS. To control the bending angle of the finger of the robotic hand, *V*_MPS_ was applied through a function generator (33220A, Agilent). The synaptic memory current generated by NeuroMAT was applied to the inverting amplifier to convert *I*_synapse_ into *V*_memory_. Therefore, *V*_memory_ was suitable for comparison with *V*_MPS_. The width of the pulse output was determined by a comparison between *V*_memory_ and *V*_MPS_ (Fig. 4C), and the resultant pulse output was applied to the servomotor of the robotic hand. Last, the fingers of the robotic hand were bent by operating the servomotor according to the width of the output pulse.

## Supplementary Materials

### This PDF file includes:

Supplementary Text Figs. S1 to S32 Table S1 Legends for movies S1 and S2 References
