# Supplementary material for: Ion trap and release dynamics enables nonintrusive tactile augmentation in monolithic sensory neuron
Source: Sci Adv. 2023 Oct 18;9(42):eadi3827. doi: 10.1126/sciadv.adi3827 (PMC10584339; doi:10.1126/sciadv.adi3827)
Supplement: Supplementary file 1 — Supplementary Text Figs. S1 to S32 Table S1 Legends for movies S1 and S2 References [file sciadv.adi3827_sm.pdf]

Science Advances
AAAS

Supplementary Materials for

Ion trap and release dynamics enables nonintrusive tactile augmentation in monolithic sensory neuron

Hyukmin Kweon *et al.*

Corresponding author: Seung Geol Lee, seunggeol.lee@pusan.ac.kr; Jeong Ho Cho, jhcho94@yonsei.ac.kr; Do Hwan Kim, dhkim76@hanyang.ac.kr

Sci. Adv. **9**, eadi3827 (2023)
DOI: 10.1126/sciadv.adi3827

The PDF file includes:

Supplementary Text
Figs. S1 to S32
Table S1
Legends for movies S1 and S2
References

Other Supplementary Material for this manuscript includes the following:

Movies S1 and S2

Supplementary Text

Theoretical background of ion dynamics

For a solution electrolyte, the ion flux (J_i) can be mathematically described by considering contributions from both ion diffusion and ion migration, and is given by the following equation;

$$J_i = -D_i \nabla c_i - z_i u_i F c_i \nabla \phi \quad \text{(S1)}$$

The first term ($D_i \nabla c_i$) represents the contribution from the ion diffusion, where D_i is the diffusion constant. The second term ($z_i u_i F c_i \nabla \phi$) corresponds to the contribution of ion migration, representing the movement of ions with an ionic charge (z_i) and mobility (u_i). Thus, assuming a chemical equilibrium state ($\nabla c_i = 0$), the early stage of the ion flux is primarily determined by the interplay between ion concentration (c_i) and the electric potential gradient ($\nabla \phi$) which are correlated with the ion migration. By employing Einstein's equation ($u_i = D_i / k_b T$), and substituting J_i into the ion flux equation, we derived the Nernst-Plank equation as follows;

$$\frac{\partial c_i}{\partial t} = -\nabla \cdot \left(-D_i \nabla c_i \mp \frac{z_i D_i e}{k_b T} c_i \nabla \phi\right) \quad \text{(S2)}$$

where e is the electronic charge, k_b is the Boltzmann constant and T is temperature. The molecular theories of ion dynamics and transport are primarily based on Poisson-Nernst-Plank (PNP) equation. From a Nernst-Plank equation, a potential distribution in an electrolyte can be determined by the Poisson equation as follows;

$$\nabla^2 \phi = -\frac{\rho}{\epsilon} = \frac{e \sum_i c_i z_i}{\epsilon} \quad \text{(S3)}$$

where ϵ is the dielectric constant of the medium and ρ is the net charge density. For a symmetrical electrolyte and assuming, we can develop Debye-Falkenhagen equation in terms of the potential as follows.

$$\frac{1}{D} \frac{\partial \phi}{\partial \epsilon} = (\nabla^2 - \kappa^2) \phi \quad \text{(S4)}$$

where κ^{-1} is the Debye screening length which is given by

$$\kappa^{-1} = \sqrt{\frac{\epsilon_r \epsilon_0 k_B T}{2 N_A e^2 I}} \quad \text{(S5)}$$

where I is the ionic strength, k_B is the Boltzmann's constant, N_A is the Avogadro's number, ϵ_r is the dielectric constant, ϵ_0 is the permittivity of free space and T is the temperature. From these equations, we can deduce that an ionic concentration is the main factor contributing to a variation of the potential distribution and the Debye screening length (corresponding to a thickness of diffuse layer in the electrolyte). For a sinusoidal applied potential ($\phi = \phi(r) e^{iwt}$), the Debye-Falkenhagen equation is described as follows (*27*);

$$[\nabla^2 - \kappa^2 (1 + iw\tau_D)] \phi(r) = 0 \quad \text{(S6)}$$

where $\tau_D = 1/\kappa^2 D$ is the Debye time and w is the frequency. We note that observation of the Debye time provides valuable insights to comprehend ion trap and release dynamics (iTRD) because the Debye time depends on an ionic concentration regardless of a dimensional change (e.g., thickness and area) of iTRD-iongel film.

A bode plot is a frequency-dependent impedance spectrum, by which dielectric relaxation times of charged species in iongel materials can be investigated empirically. As shown in Fig. 2H and fig. S8B, Debye relaxation frequency ($\omega_D = {\tau_D}^{-1}$) can be extracted from an intersection point of real part (Z') and imaginary part (Z'') impedance spectra in Bode plots (*45*). According to the Debye-Falkenhagen relation (Eq. S6), a shift of Debye relaxation frequency toward higher frequencies means a shorter Debye screening length caused by an increase of ionic concentration in iongel materials. Based on this theoretical interpretation, the higher frequency shift of the Debye relaxation frequency (decreased Debye time) under pressure can be regarded as an increase of ionic concentration in the iTRD-iongel (Fig. 2H), which was attributed to the release of trapped ions.

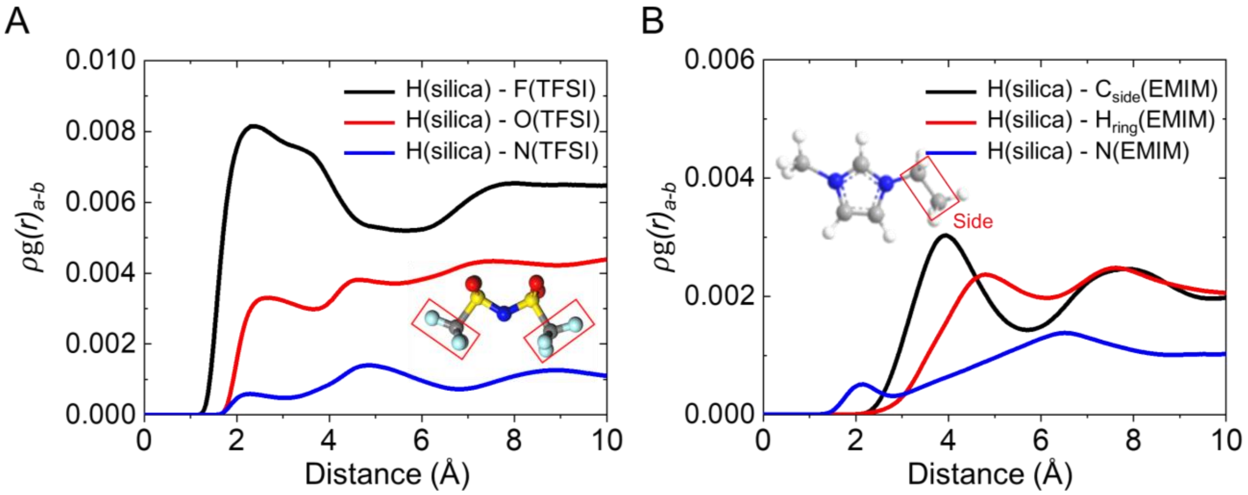

Fig. S1. Pair correlation function (PCF) of hydrogen on the top of silica surface and the components of (A) [TFSI]$^-$ and (B) [EMIM]$^+$, respectively. The $\rho g(r)_{a\text{-}b}$ of each atomic pairs was utilized for direct comparison of atomic distribution, which is determined by calculating the probability of finding 'a' atom around 'b' atom separated by interatomic distance over the equilibrium structure (a: Hydrogen atom of silica, b: Carbon, hydrogen, fluorine, nitrogen, and oxygen atoms of [EMIM]$^+$ and [TFSI]$^-$).

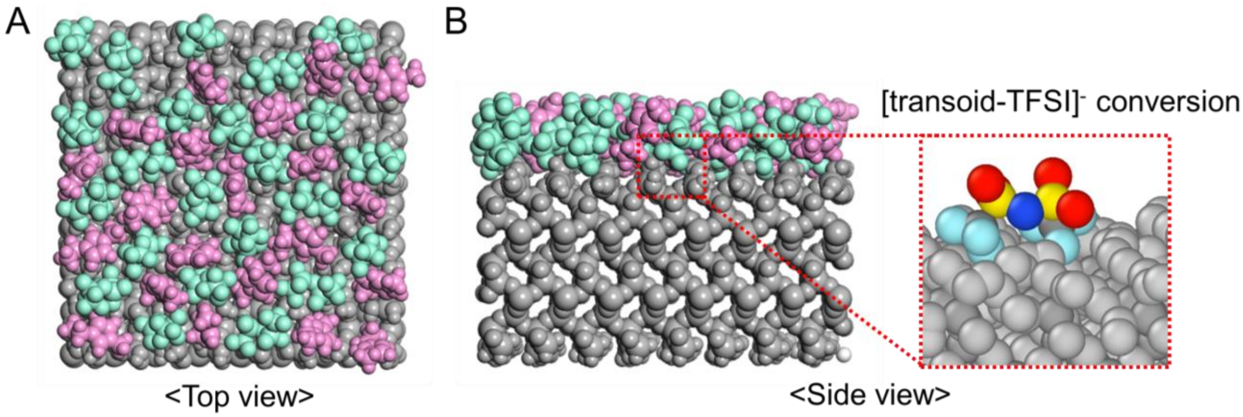

Fig. S2. Snapshot of the MD simulation of $[EMIM]^+[TFSI]^-$ on the surface of silica particles. (**A**) the first adsorption layer (top view) and (**B**) equilibrium configuration (side view) of $[EMIM]^+[TFSI]^-$ on the silica particle surface.

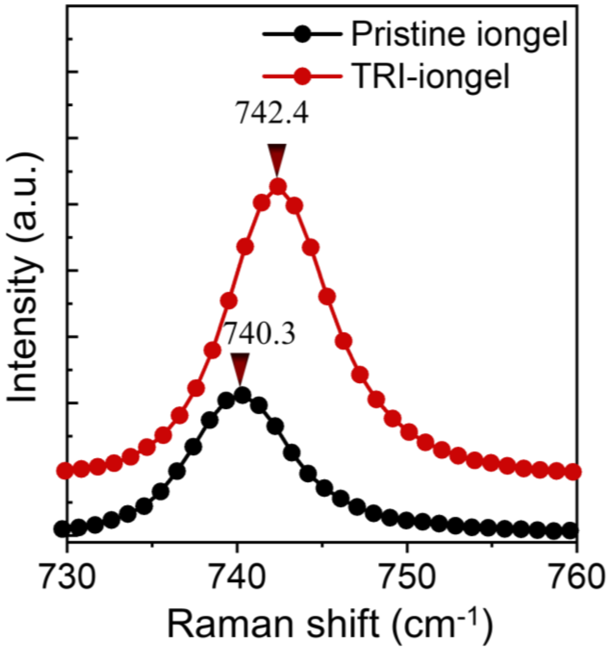

Fig. S3. Raman spectra in the spectral range 730–760 cm^{-1}. The raman spectral range is corresponding to expansion-contraction mode of $[TFSI]^{-}$.

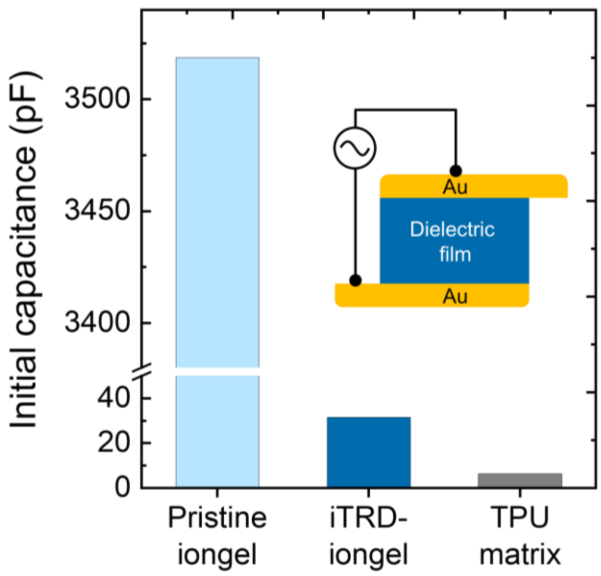

Fig. S4. Evaluation of initial capacitance of pristine iongel-, iTRD-iongel, and TPU matrix-based capacitors. The initial capacitance was measured at 1V @ 1 kHz, and the thickness of the three films were 200 µm.

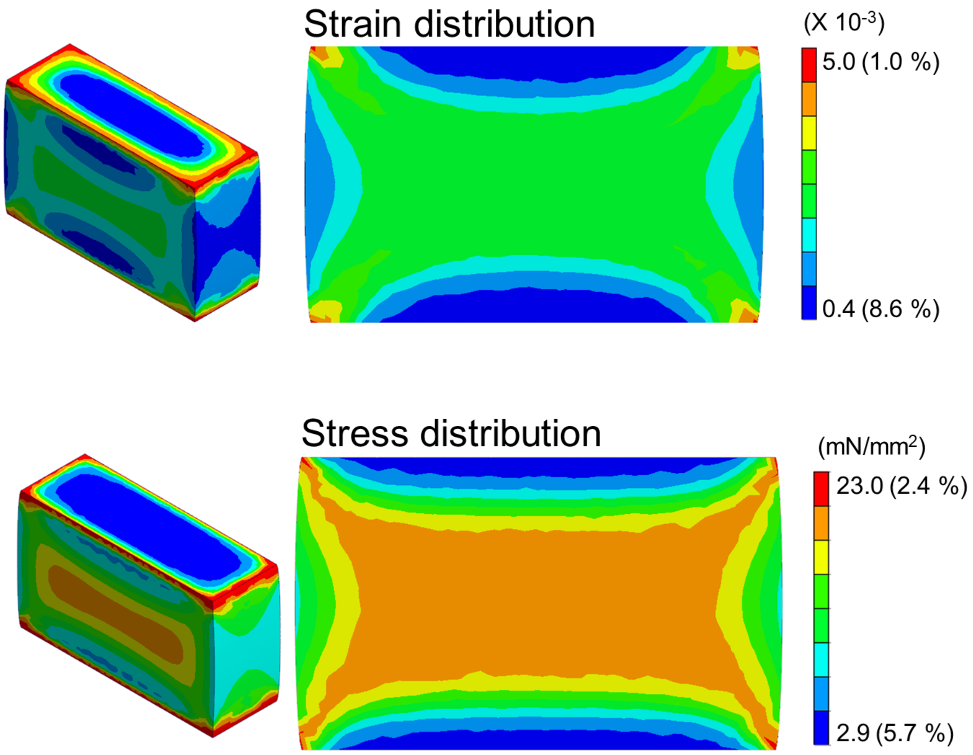

Fig. S5. Finite element method (FEM) calculation of the pristine iongel. The effective stress and strain within the pristine iongel were exhibited when pressure was applied.

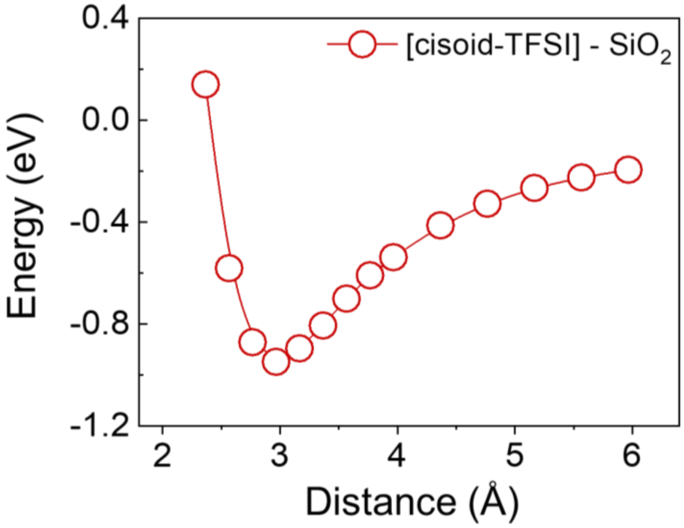

Fig. S6. Binding energy curves as a function of distance between silica surface and [cisoid-TFSI] by DFT calculations.

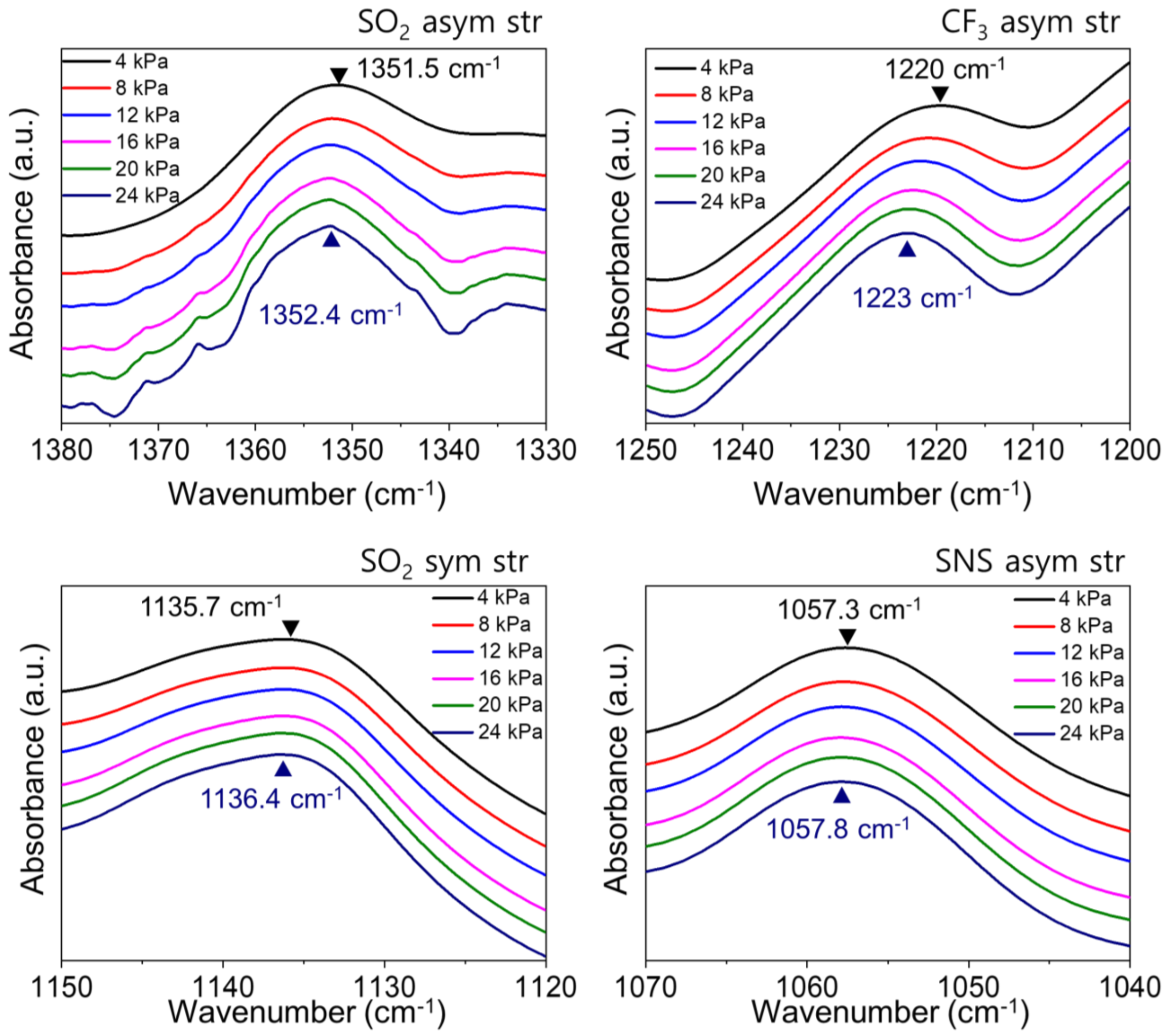

Fig. S7. FTIR spectra of [TFSI]$^-$ in the spectral ranges of 1330–1380 cm^{-1}, 1200–1250 cm^{-1}, 1120–1150 cm^{-1}, and 1040–1070 cm^{-1}, corresponding to [TFSI]$^-$ vibrational bands. Shifting of FTIR vibrational bands of SO_2, CF_3, and SNS groups of [TFSI]$^-$ toward higher wavenumbers under pressure clearly indicated that [TFSI]$^-$ possessed fewer interactions (reduced degree of freedom in molecular motion) with surrounding molecules over silica particles due to cleavage of hydrogen bonds with the silanol groups.

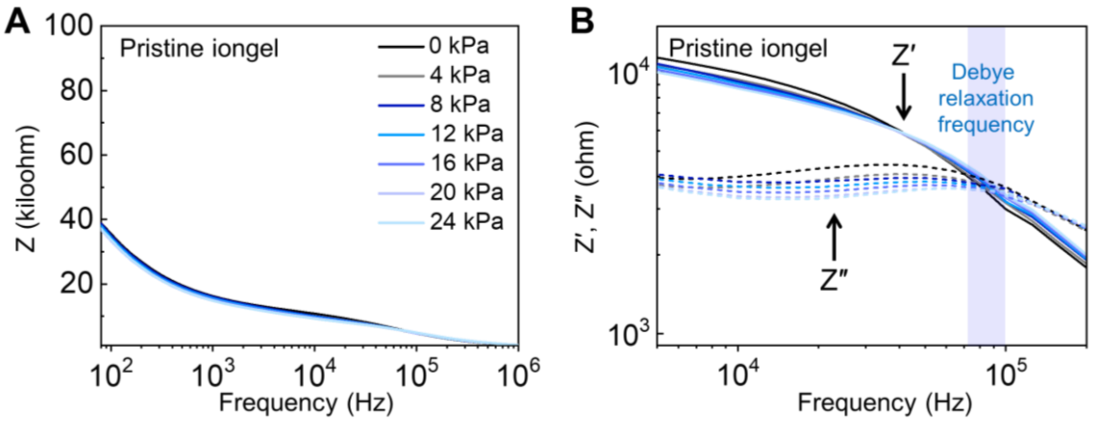

Fig. S8. Bode plots for pristine iongel under various applied pressures. (**A**) Frequency-dependent complex impedance spectra of pristine iongel under various pressures (0–24 kPa). (**B**) Frequency-dependent real (solid line) and imaginary (dashed line) impedance spectra of the pristine iongel under various pressures (0–24 kPa).

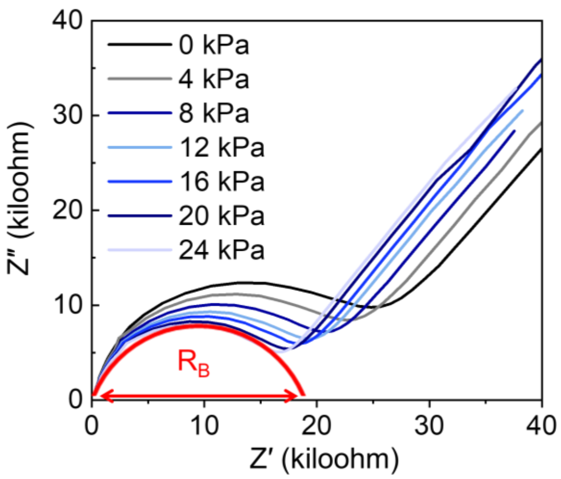

Fig. S9. Nysquist plot of iTRD-iongel in response to various pressures (0–24 kPa). The ionic conductivity of iTRD-iongel enhanced from 0.0031 mS/cm to 0.0053 mS/cm as the applied pressure increased from 0 kPa to 24 kPa.

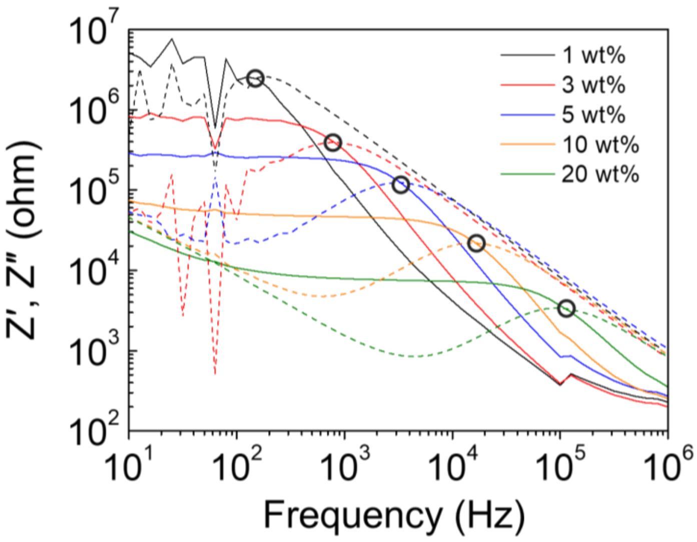

Fig. S10. Frequency-dependent real (solid line) and imaginary (dashed line) impedance spectra of pristine iongel with different ionic concentration.

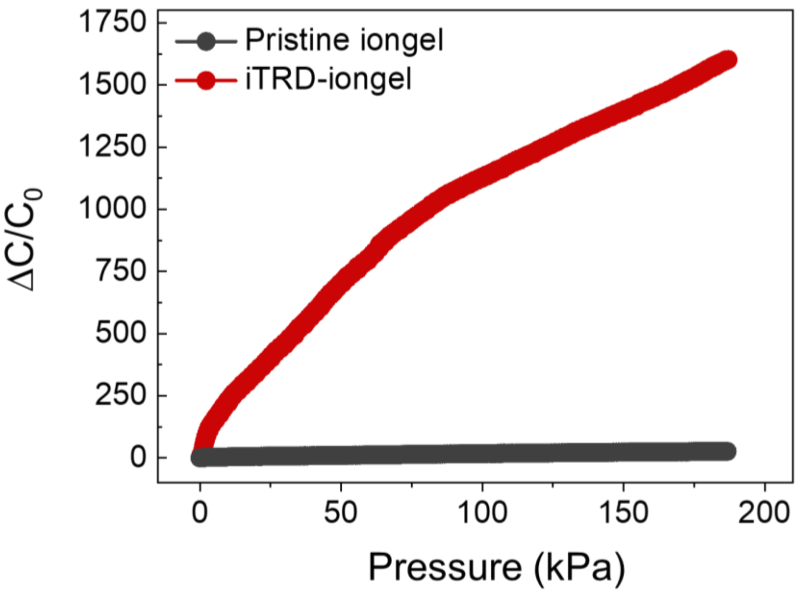

Fig. S11. Comparison of capacitance change between the pristine and iTRD-iongel as a function of applied pressure. The ΔC is given by C_p-C_0 where C_p and C_0 are a pressure-modulated and an initial capacitance values, respectively.

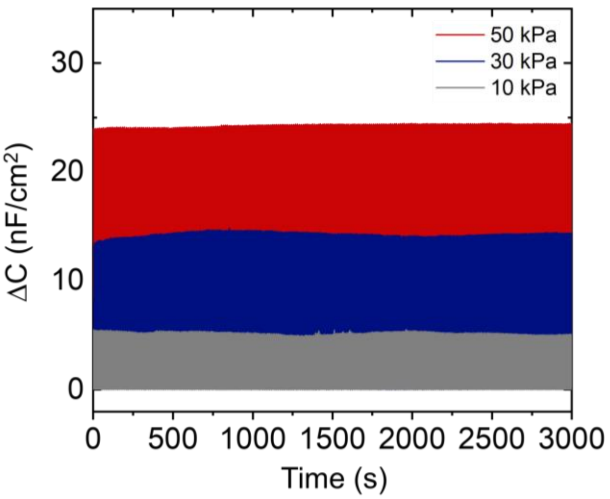

Fig. S12. Evaluation of response durability of iTRD-iongel. The ΔC was measured from iTRD-ion-based capacitor with 300 cycles of applied pressure (10, 30, and 50 kPa) under 1V @ 100 Hz.

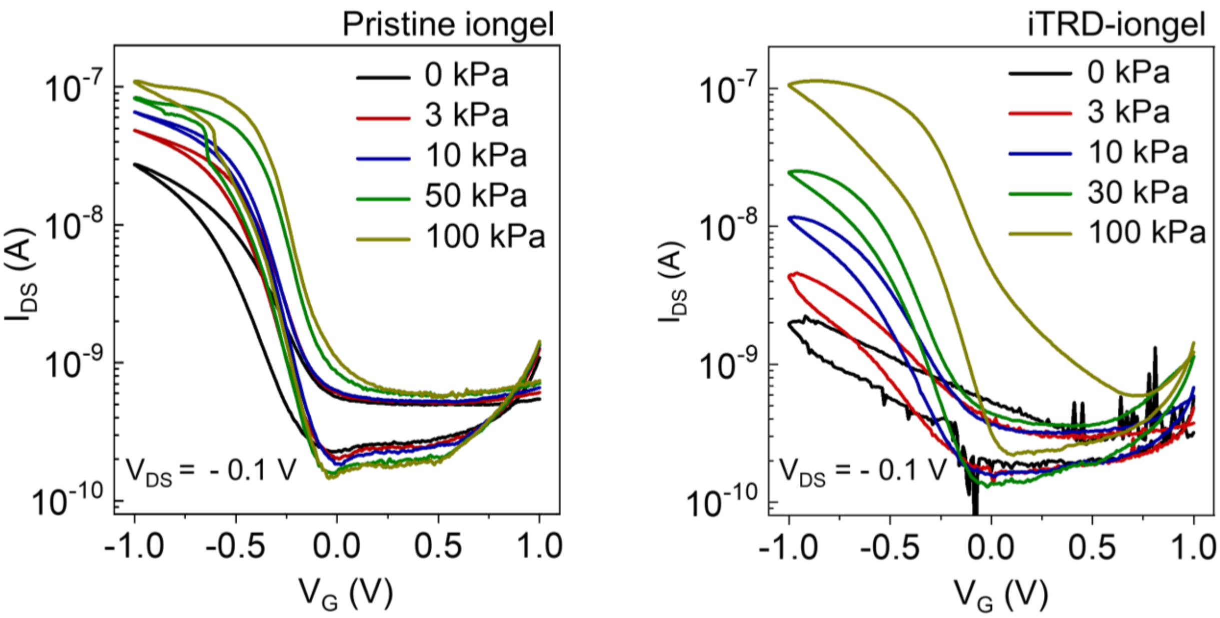

Fig. S13. Transfer characteristics of pristine iongel-based synaptic transistor and iTRD-driven NeuroMAT. The electrical properties were evaluated depending on the applied mechanical stimuli from 0 kPa to 100 kPa.

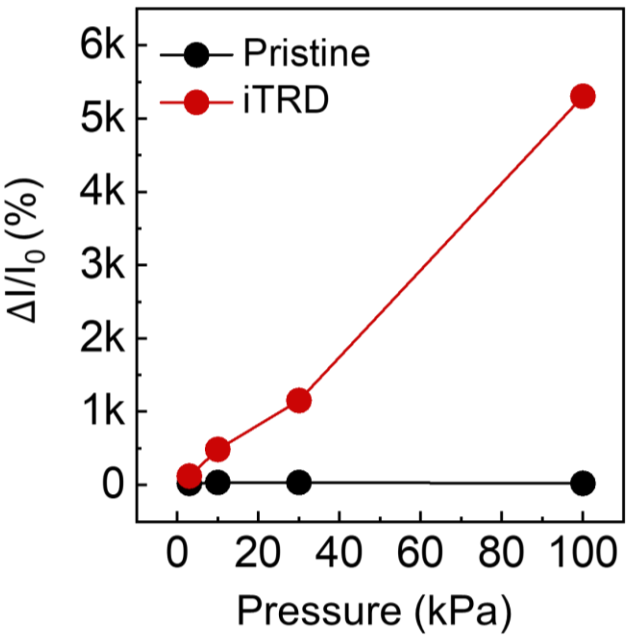

Fig. S14. Comparison of pressure sensitivity of pristine iongel-based synaptic transistor and iTRD-driven NeuroMAT.

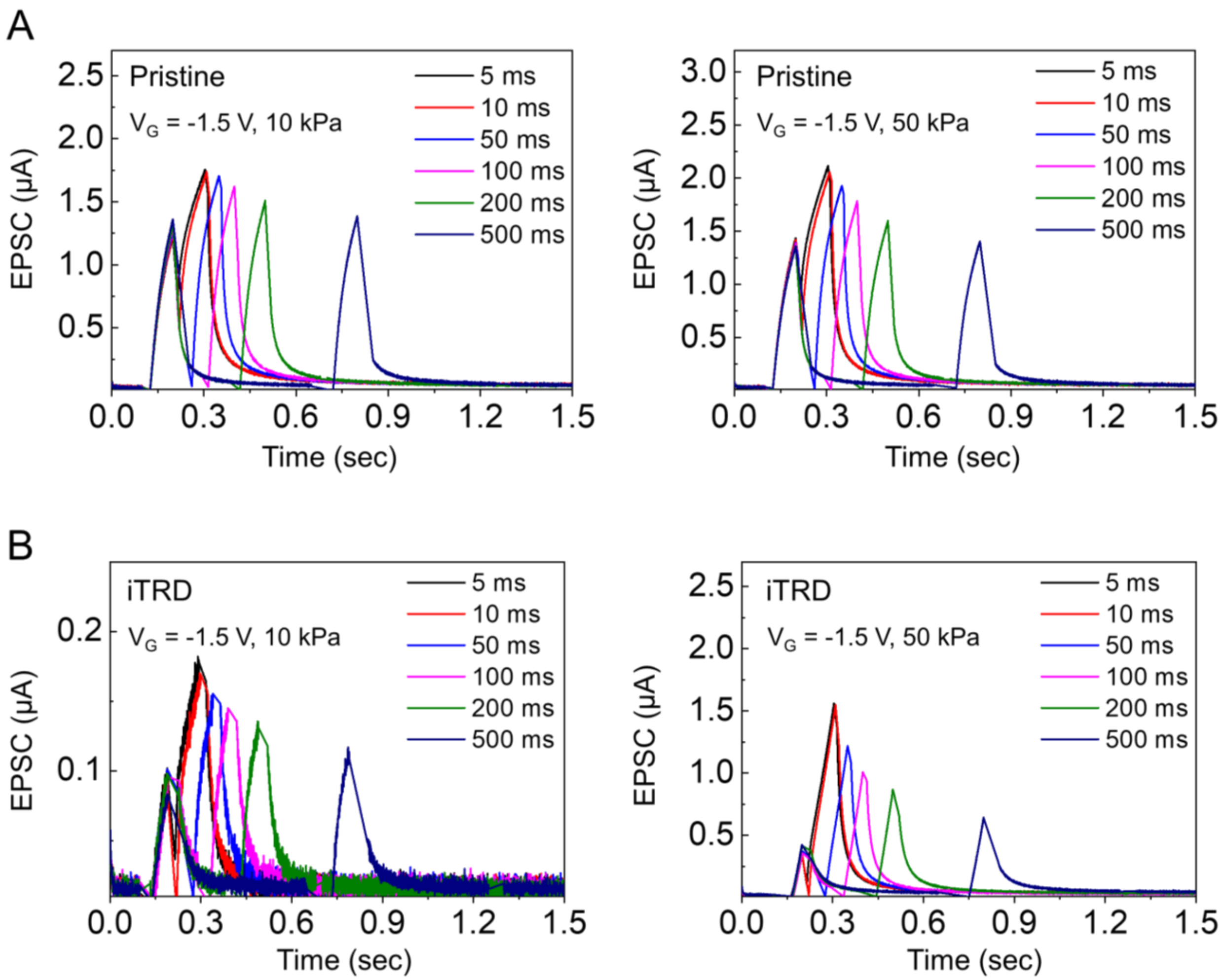

Fig. S15. Analysis of excitatory postsynaptic current (EPSC). The EPSC of (**A**) pristine iongel-based synaptic transistor and (**B**) iTRD-driven NeuroMAT was measured when mechanical stimuli (10 kPa and 50 kPa) were applied.

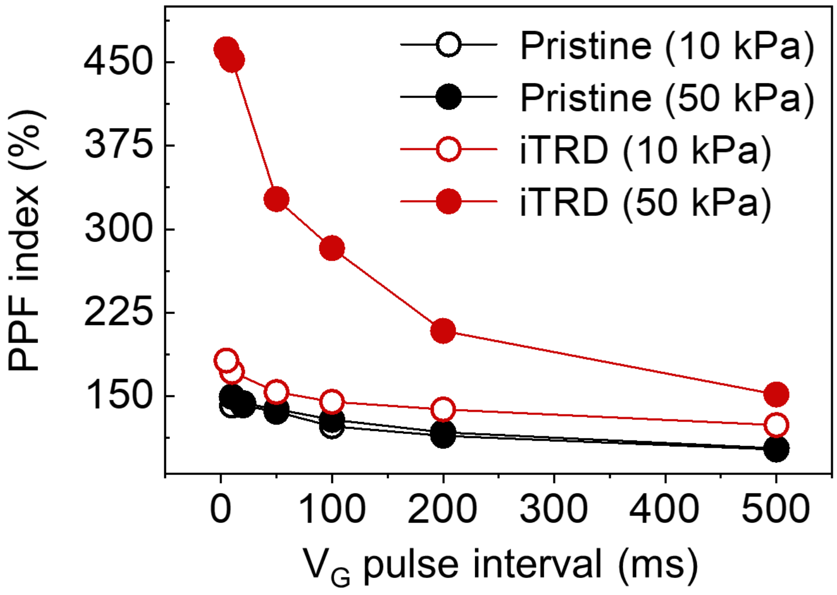

Fig. S16. Comparison of paired-pulse facilitation (PPF) of pristine iongel-based synaptic transistor and iTRD-driven NeuroMAT under different pressure (10 kPa and 50 kPa).

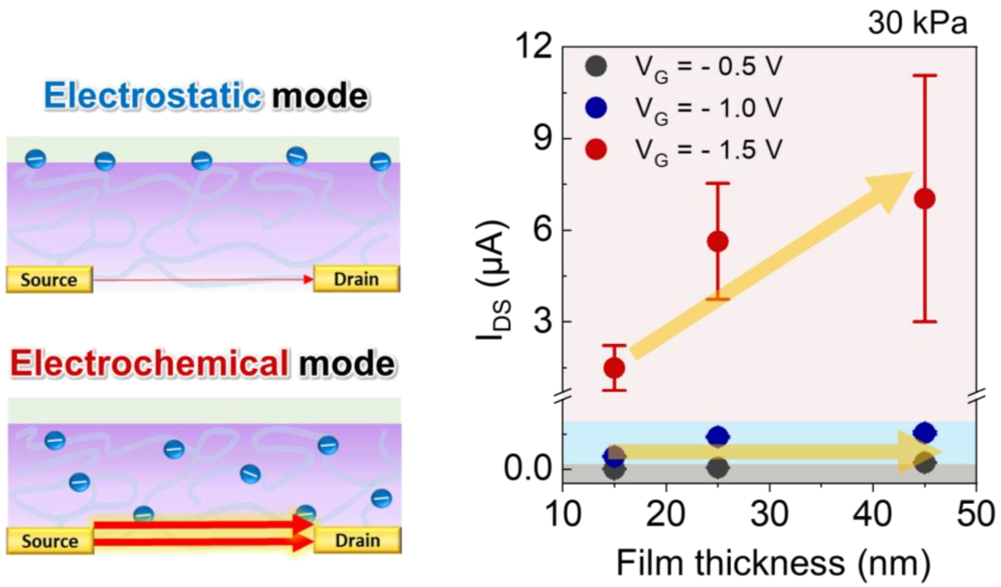

Fig. S17. Variation of the drain current of iTRD-driven NeuroMAT as a function of PDPPTT films with different V_G. Ion penetration within the PDPPTT layer of the NeuroMAT was clearly observable when -1.5 V of V_G was applied.

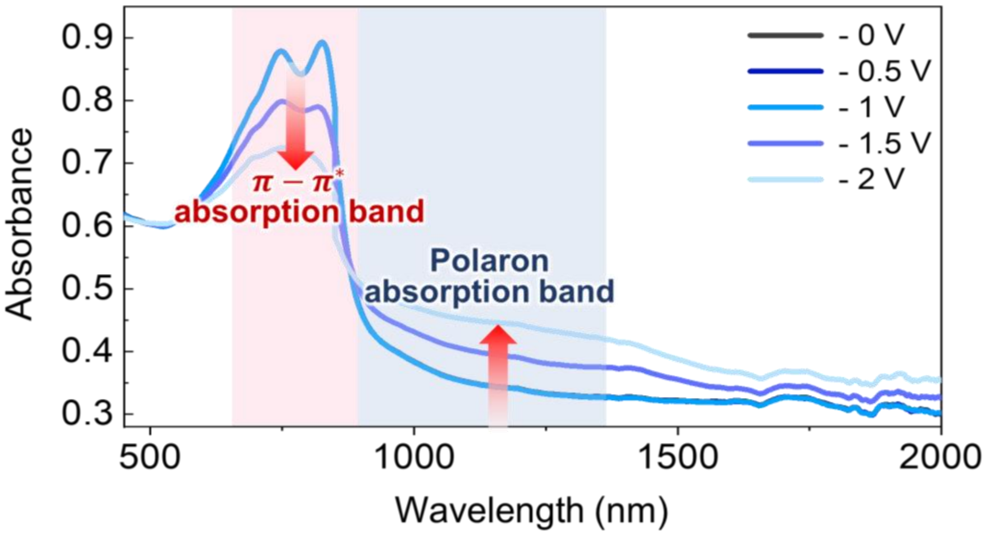

Fig. S18. UV-vis absorption spectra of PDPPTT with $[EMIM]^{+}[TFSI]^{-}$. The variation of the absorption spectra was obtained under applied voltage ranging from 0 V to -2 V.

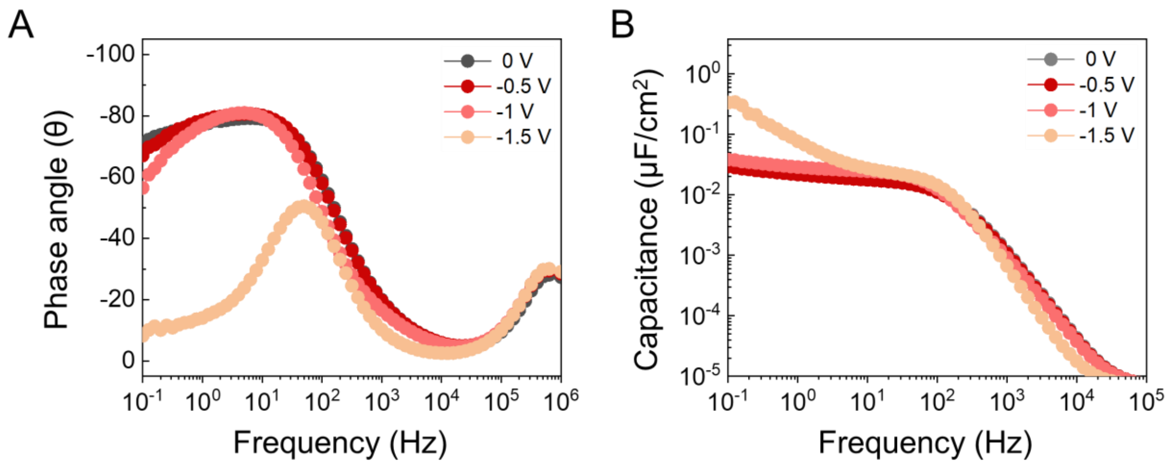

Fig. S19. (**A** and **B**) **Phase angle and capacitance plots as a function of frequency for a capacitor with PDTTPP/iTRD-iongel.** The phase behavior of the impedance spectra was analyzed by applying the different DC voltage with a rms amplitude of 10 mV under pressure. The capacitance was extracted from the complex impedance in an equivalent circuit which consists of a resistor and a capacitor in parallel.

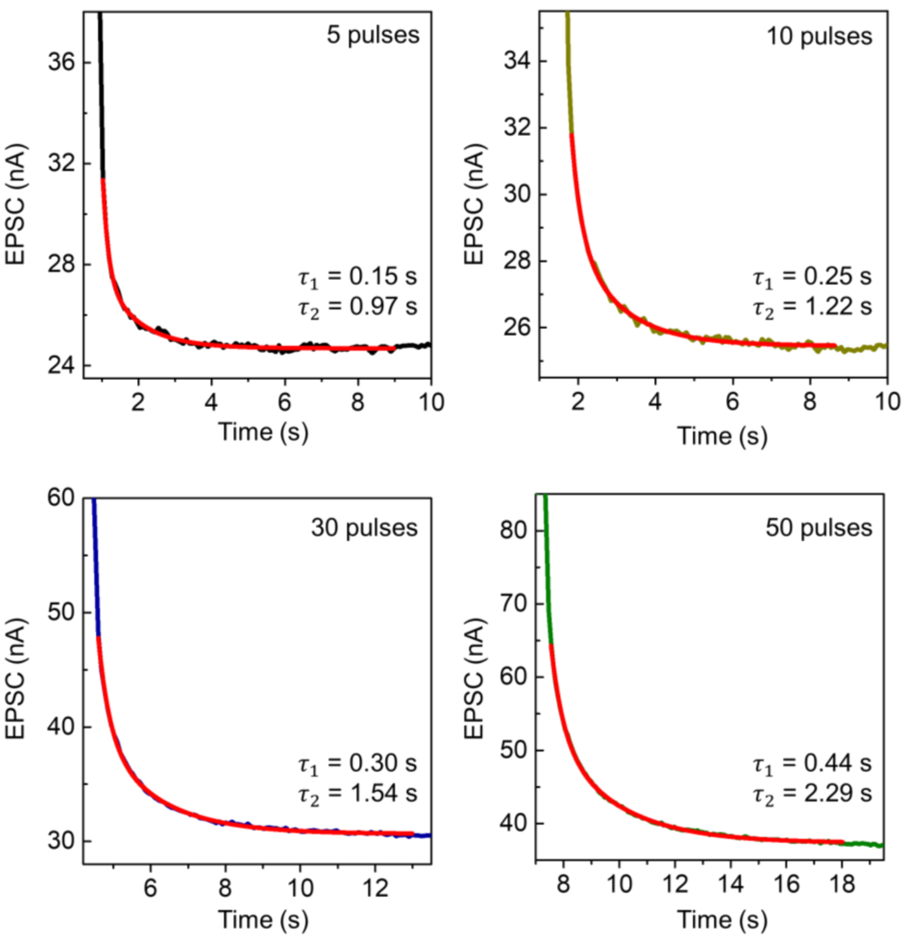

Fig. S20. EPSC decay characteristics of iTRD-driven NeuroMAT depending on the number of V_G pulses. The EPSCs were fitted (red lines) and the two decay times ($\boldsymbol{\tau_1}$ and $\boldsymbol{\tau_2}$) were extracted by bi-exponential decay model ($\boldsymbol{I_{EPSC} = I_0 + I_1 e^{-t/\tau_1} + I_2 e^{-t/\tau_2}}$, $\boldsymbol{\tau_1}$ and $\boldsymbol{\tau_2}$ are corresponding to depolarization of EDL and anion de-doping time, respectively) (*31*).

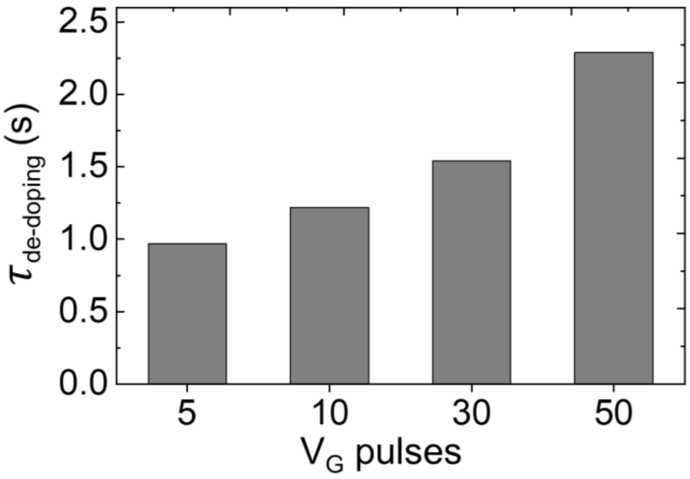

Fig. S21. The calculated anion de-doping times ($\tau_{de-doping}$) as a function of applied V_G pulses.

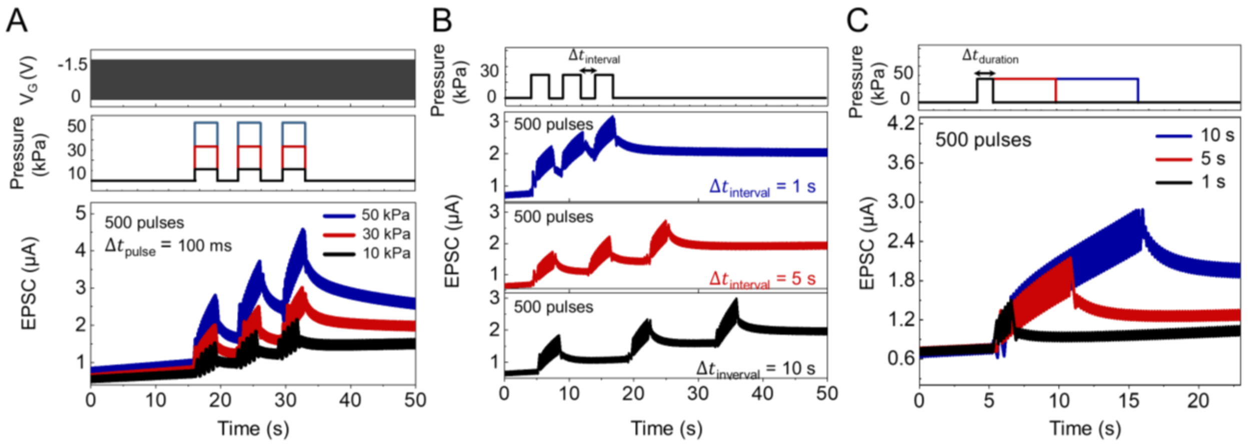

Fig. S22. Tactile perception performance of iTRD-driven NeuroMAT toward dynamic mechanical stimuli under a series of V_G pulses, corresponding to (A) amplitude of pressure, (B) frequency, and (C) duration time.

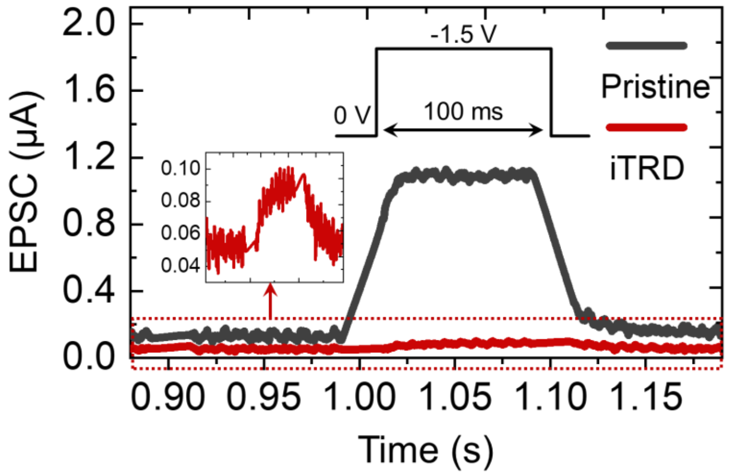

Fig. S23. Comparison of response of excitatory postsynaptic current (EPSC) signals between pristine iongel-based synaptic transistors and NeuroMAT toward a single V_G pulse.

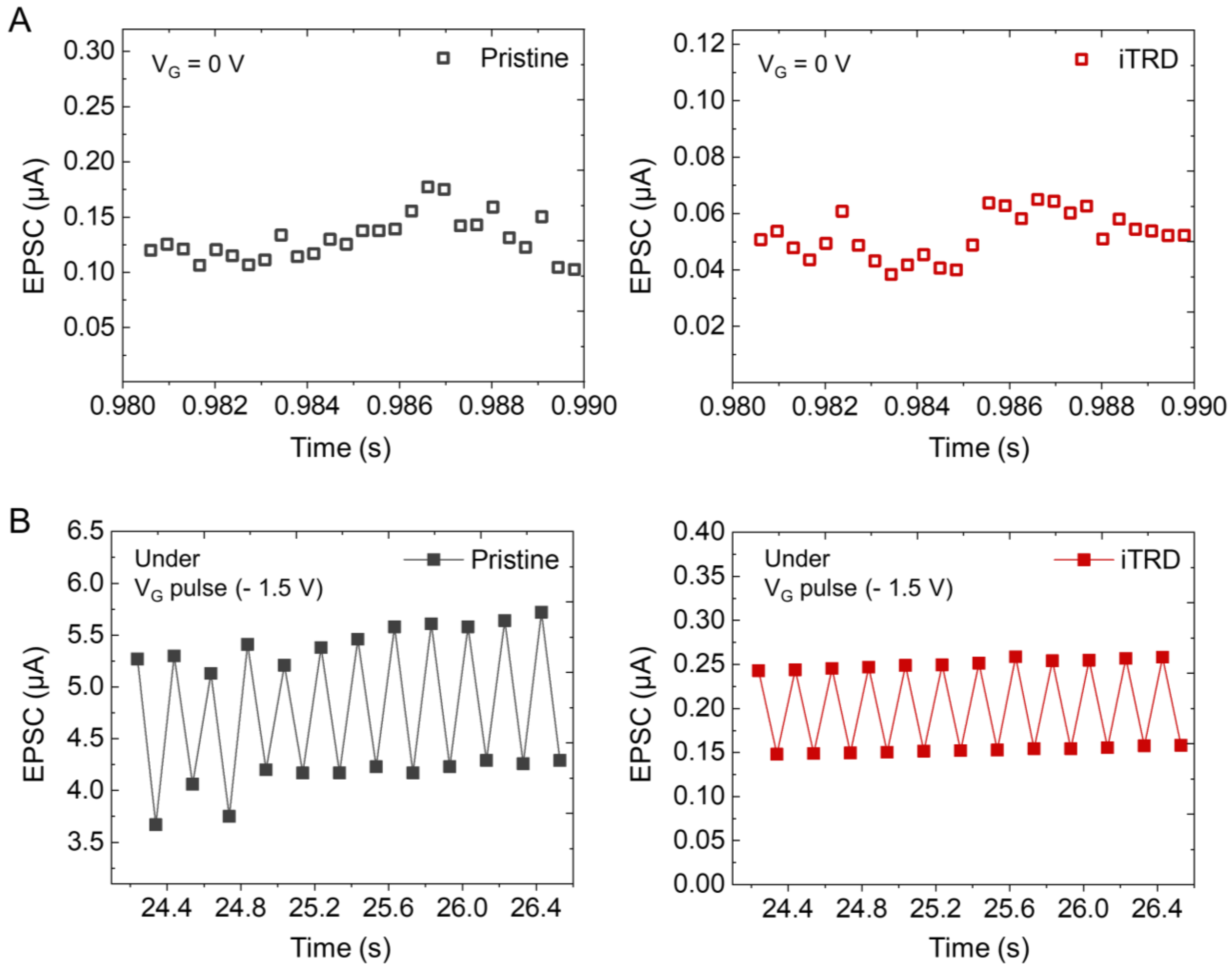

Fig. S24. Collection of background noise currents for signal-to-noise ratio (SNR) calculation. The collection of baseline current points of pristine iongel-based synaptic transistors and iTRD-driven NeuroMAT (**A**) without V_G and (**B**) with V_G pulses, which were extracted from fig. S23 and Fig. 2B, respectively. The detailed calculation method was followed by the previous studies (*29*, *30*).

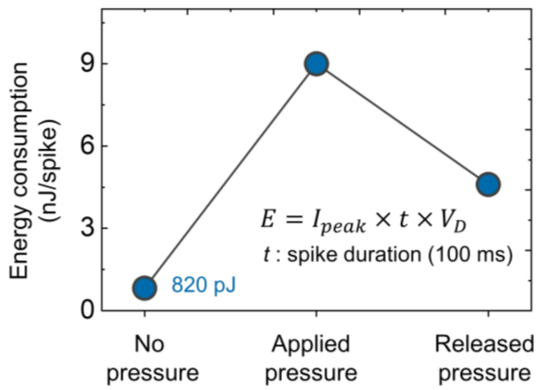

Fig. S25. Energy consumption of NeuroMAT depending on pressure under continuous V_G stimuli. The calculated energy consumption of NeuroMAT was extracted from Fig. 3B.

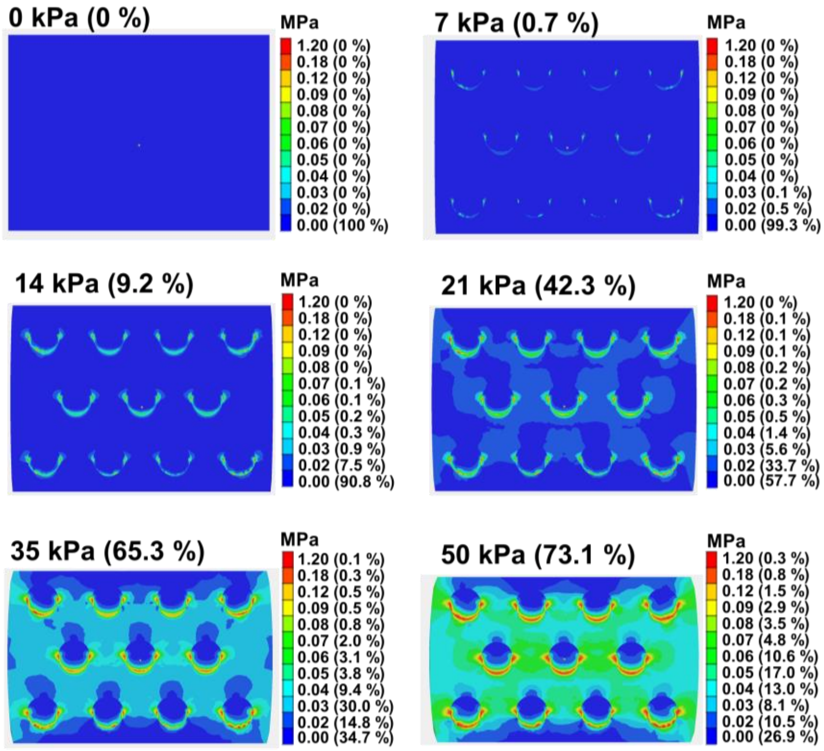

Fig. S26. FEM simulation showing the magnitude and the distribution of stresses within iTRD-iongel under different pressures (0–50 kPa).

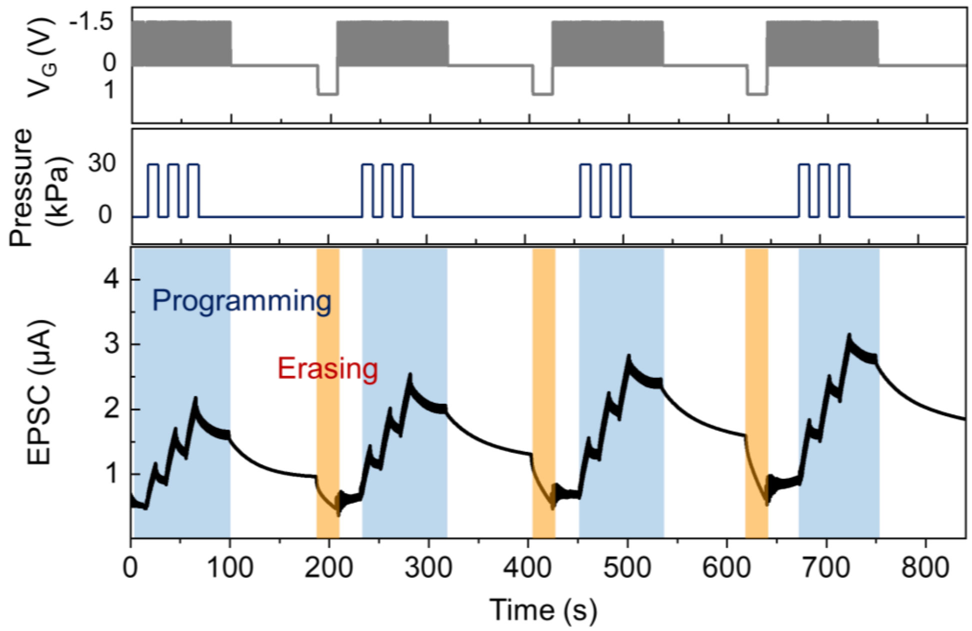

Fig. S27. Programming and erasing cycles of tactile memory of NeuroMAT.

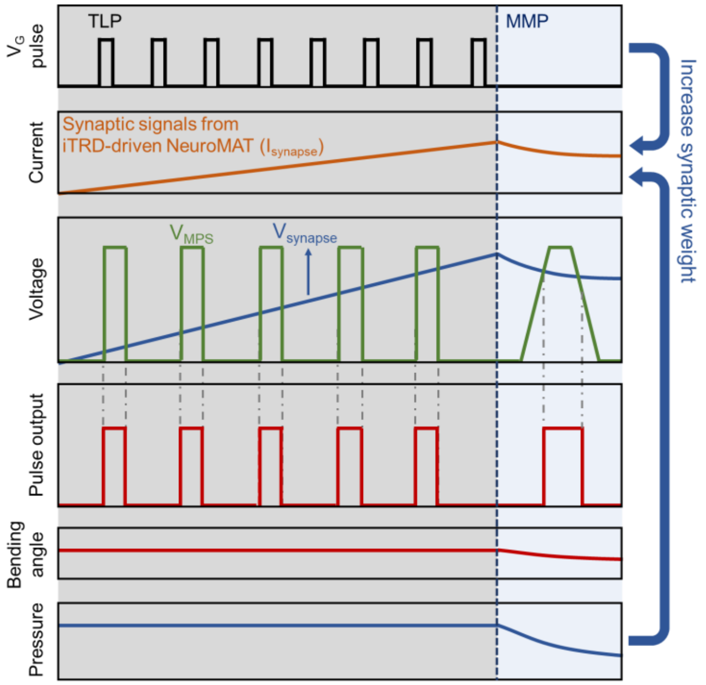

Fig. S28. Detailed schematic diagram of TLP in NeuroMATICS. During TLP process, the square waveform of V_G and V_{MPS} pulses were applied to iTRD-driven NeuroMAT and motor position system (MPS), respectively (Fig. 4A). As anthropomorphic robotic hand grabbed the object, pressure was induced on the NeuroMAT which was attached on the robotic finger. Synaptic signals ($I_{synapse}$) was increased due to the applied V_G pulses and the pressure. In the case of the TLP, the width of the pulse output did not change due to the square waveform of V_{MPS}.

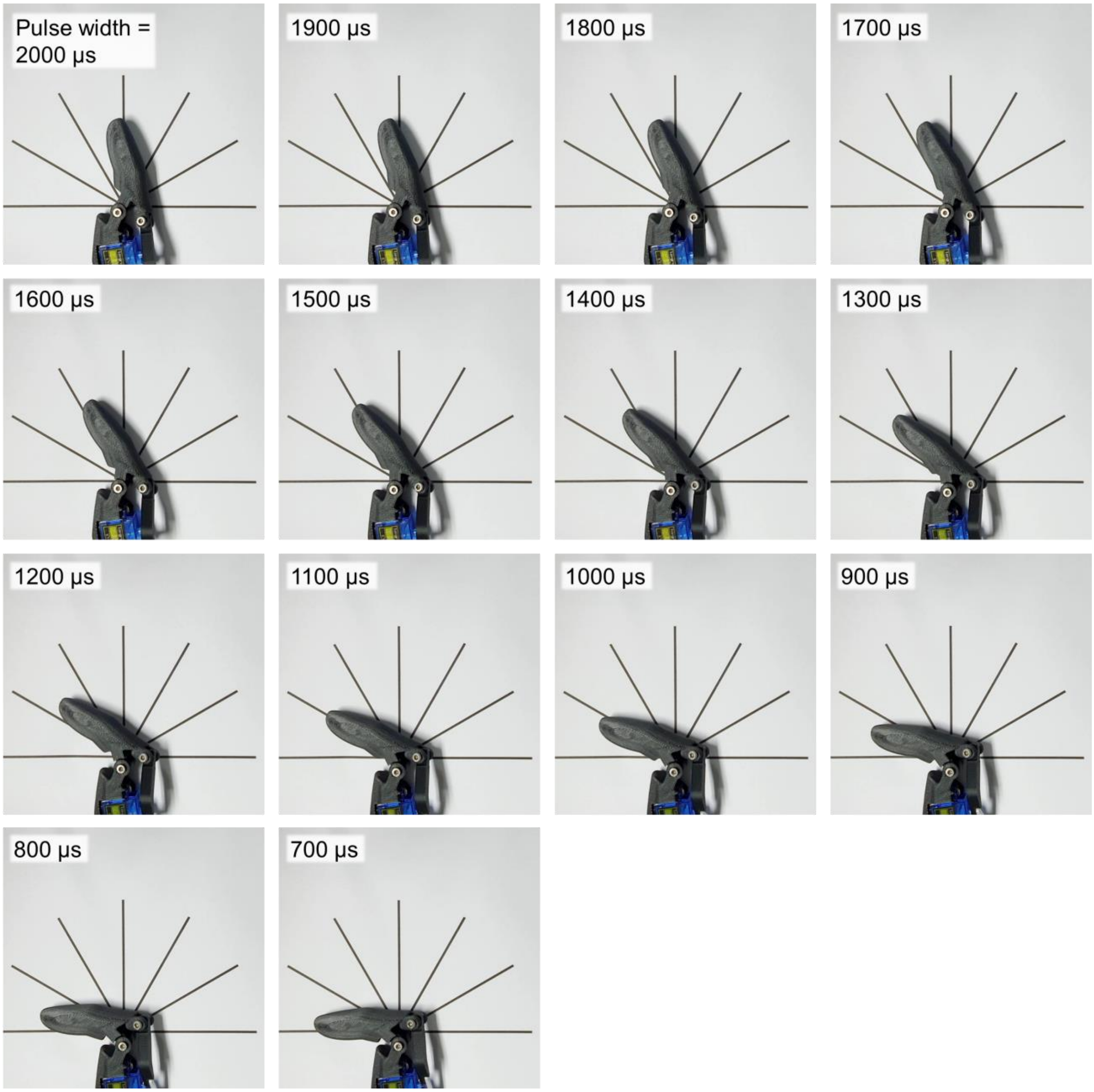

Fig. S29. Photograph of bending motion of the robotic hand. The bending angle was determined by the width of pulse output.

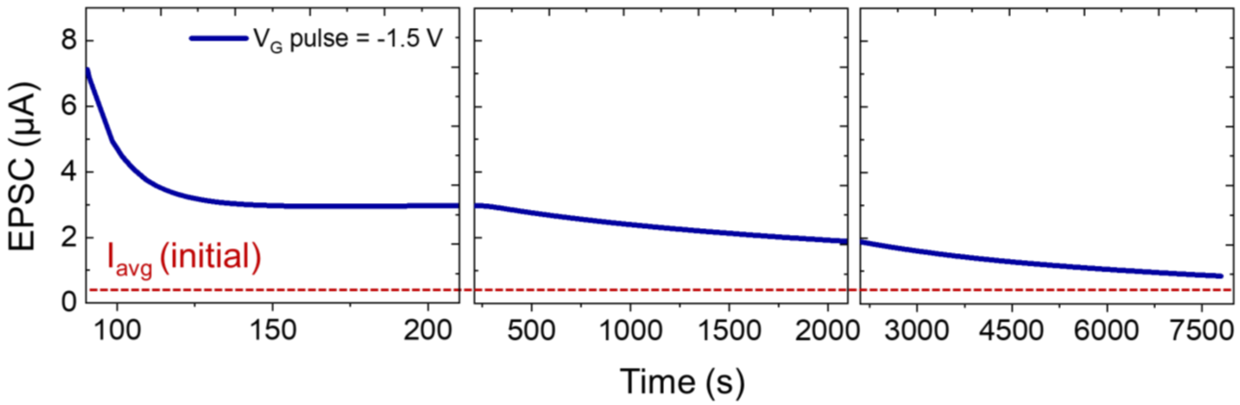

Fig. S30. Long-term tactile memory characteristics of iTRD-driven NeuroMAT. The tactile memory induced by a single-trial learning was maintained over 7500 s.

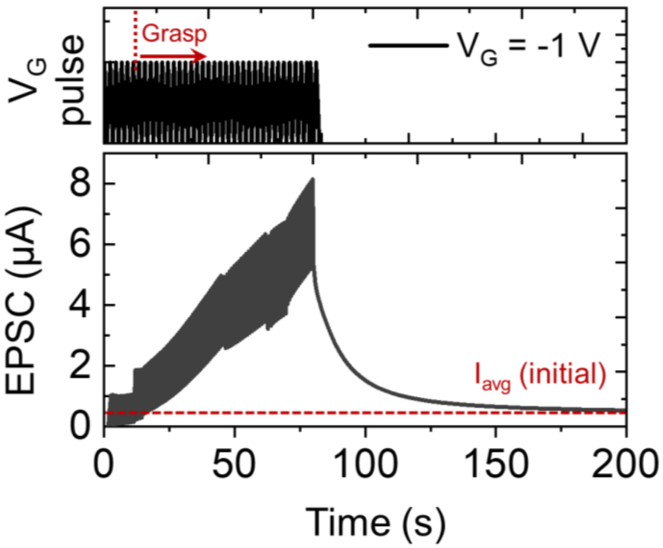

Fig. S31. Non-tactile memory characteristics of iTRD-driven NeuroMAT.

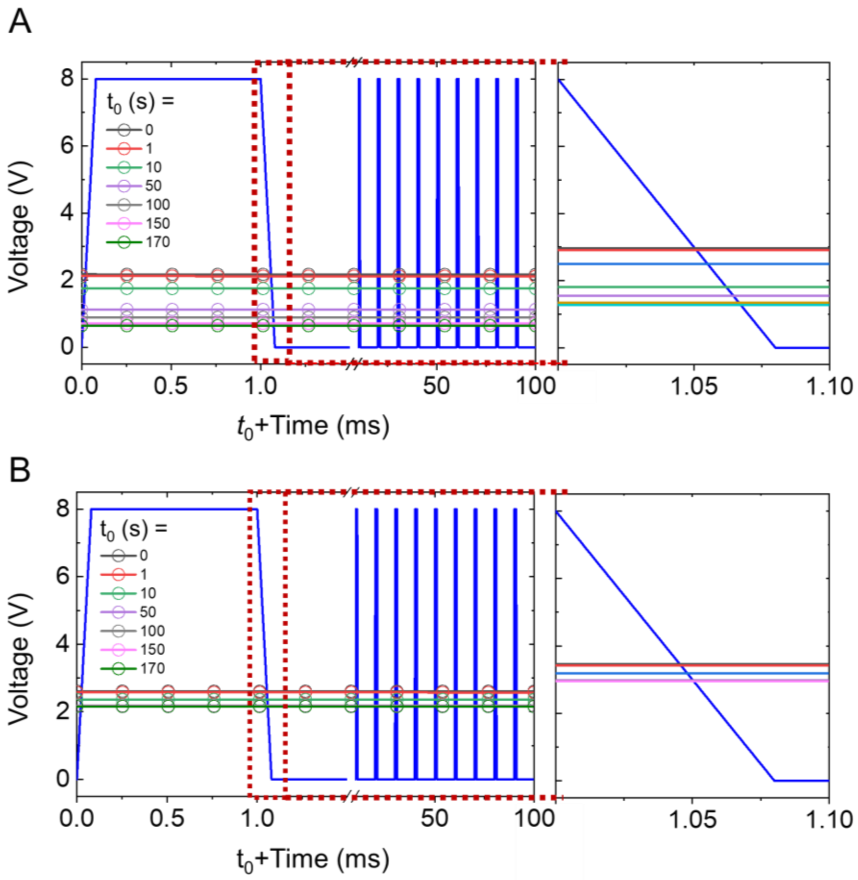

Fig. S32. Comparison of V_{memory} and V_{MPS} for (A) non-tactile memory and (B) tactile memory situation. The solid line (blue) and line with symbols represented V_{MPS} and V_{memory}, respectively. The width of pulse output increased when the V_{memory} decreased as a function of time.

System	Ionic materials	Operating voltage	Retention time of LTM	Ref.
Our work	TPU+Silica microparticles /$[EMIM]^{+}[TFSI]^{-}$	-1.5 V	> 3680 s	
Additional transition gate	P(VDF-HFP)/$[EMIM]^{+}[TFSI]^{-}$	-1 V	> 200 s	(*19*)
Dual-connected synaptic transistors	Chitosan	-10 V	N/A	(*46*)
Integration of tactile sensor & synaptic transistor	(PS-b-PEO-b-P)/$[EMIM]^{+}[TFSI]^{-}$	< -2 V	N/A	(*15*)
Integration of tactile sensor & synaptic transistor	PVA Hydrogel	< -1 V	N/A	(*16*)
Integration of tactile sensor & synaptic transistor	(PS-PMMA-PS)/$[EMIM]^{+}[TFSI]^{-}$	-2.5 V	> 5 s	(*47*)
Integration of tactile sensor & synaptic transistor	PVA Hydrogel	-1 V	N/A	(*17*)
Integration of tactile sensor & synaptic transistor	$[Li]^{+}[TFSI]^{-}$	< 13 V	> 20 s	(*48*)
Integration of tactile sensor & synaptic transistor	$[EMIM]^{+}[TFSI]^{-}$	< -2 V	N/A	(*18*)
Integration of tactile sensor & synaptic transistor	PVA/Malic acid	< -3 V	100 s	(*49*)
Integration of tactile sensor & synaptic transistor	PAN/$[Li]^{+}[TFSI]^{-}$	-8 V	N/A	(*50*)
Integration of tactile sensor & synaptic transistor	Chitosan	< 5 V	5 s	(*51*)
Integration of tactile sensor & synaptic transistor	P(VDF-HFP)/$[EMIM]^{+}[TFSI]^{-}$	-3 V	> 7 s	(*52*)

Table S1. Comparison of the tactile memory performance of previously reported artificial tactile nerve systems.

Movie S1. A cisoid configuration of $[TFSI]^{-}$ on a surface of silica microparticles.

Movie S2. Grip motion of robotic hand integrated with NeuroMAT. Movie S2 showed a comparison of non-tactile memory (left) and augmented tactile memory (right)-based grip motions of NeuroMATICS.

REFERENCES AND NOTES

1. A. Gallace, C. Spence, The cognitive and neural correlates of tactile memory. *Psychol. Bull.* **135**, 380–406 (2009).

2. S. Ballesteros, J. M. Reales, D. Manga, Implicit and explicit memory for familiar and novel objects presented to touch. *Psicothema* **11**, 785–800 (1999).

3. A. M. Gordon, G. Westling, K. J. Cole, R. S. Johansson, Memory representations underlying motor commands used during manipulation of common and novel objects. *J. Neurophysiol.* **69**, 1789–1796 (1993).

4. F. Liu, S. Deswal, A. Christou, Y. Sandamirskaya, M. Kaboli, R. Dahiya, Neuro-inspired electronic skin for robots. *Sci. Robot.* **7**, eabl7344 (2022).

5. D. L. Schacter, Implicit memory: History and current status. *J. Exp. Psychol. Learn. Mem. Cogn.* **13**, 501–518 (1987).

6. R. Salgado, F. Bellas, P. Caamano, B. Santos-Diez, R. Duro, A procedural long term memory for cognitive robotics, in *Proceedings of the IEEE Conference on Evolving and Adaptive Intelligent Systems*, Madrid, Spain, 17 and 18 May 2012 (IEEE, 2012), pp. 57–62.

7. M. Kaboli, G. Cheng, Robust tactile descriptors for discriminating objects from textural properties via artificial robotic skin. *IEEE Trans. Robot.* **34**, 985–1003 (2018).

8. Y. R. Lee, T. Q. Trung, B.-U. Hwang, N.-E. Lee, A flexible artificial intrinsic-synaptic tactile sensory organ. *Nat. Commun.* **11**, 2753 (2020).

9. C. Wu, T. W. Kim, J. H. Park, B. Koo, S. Sung, J. Shao, C. Zhang, Z. L. Wang, Self-powered tactile sensor with learning and memory. *ACS Nano* **14**, 1390–1398 (2020).

10. K. Lee, S. Jang, K. L. Kim, M. Koo, C. Park, S. Lee, J. Lee, G. Wang, C. Park, Artificially intelligent tactile ferroelectric skin. *Adv. Sci.* **7**, 2001662 (2020).

11. Y. Lee, H.-L. Park, Y. Kim, T.-W. Lee, Organic electronic synapses with low energy consumption. *Joule* **5**, 794–810 (2021).

12. C. S. Yang, D.-S. Shang, N. Liu, E. J. Fuller, S. Agrawal, A. A. Talin, Y.-Q. Li, B.-G. Shen, Y. Sun, All-solid-state synaptic transistor with ultralow conductance for neuromorphic computing. *Adv. Funct. Mater.* **28**, 1804170 (2018).

13. H. Wei, R. Shi, L. Sun, H. Yu, J. Gong, C. Liu, Z. Xu, Y. Ni, J. Xu, W. Xu, Mimicking efferent nerves using a graphdiyne-based artificial synapse with multiple ion diffusion dynamics. *Nat. Commun.* **12**, 1068 (2021).

14. J. Chen, Z. Zhou, B. J. Kim, Y. Zhou, Z. Wang, T. Wan, J. Yan, J. Kang, J.-H. Ahn, Y. Chai, Optoelectronic graded neurons for bioinspired in-sensor motion perception. *Nat. Nanotechnol.* **18**, 882–888 (2023).

15. Y. Kim, A. Chortos, W. Xu, Y. Liu, J. Y. Oh, D. Son, J. Kang, A. M. Foudeh, C. Zhu, Y. Lee, S. Niu, J. Liu, R. Pfattner, Z. Bao, T.-W. Lee, A bioinspired flexible organic artificial afferent nerve. *Science* **360**, 998–1003 (2018).

16. C. Wan, G. Chen, Y. Fu, M. Wang, N. Matsuhisa, S. Pan, L. Pan, H. Yang, Q. Wan, L. Zhu, X. Chen, An artificial sensory neuron with tactile perceptual learning. *Adv. Mater.* **30**, 1801291 (2018).

17. C. Wan, P. Cai, X. Guo, M. Wang, N. Matsuhisa, L. Yang, Z. Lv, Y. Luo, X. J. Loh, X. Chen, An artificial sensory neuron with visual-haptic fusion. *Nat. Commun.* **11**, 4602 (2020).

18. J. Yu, G. Gao, J. Huang, X. Yang, J. Han, H. Zhang, Y. Chen, C. Zhao, Q. Sun, Z. L. Wang, Contact-electrification-activated artificial afferents at femtojoule energy. *Nat. Commun.* **12**, 1581 (2021).

19. D. W. Kim, J. C. Yang, S. Lee, S. Park, Neuromorphic processing of pressure signal using integrated sensor-synaptic device capable of selective and reversible short- and long-term plasticity operation. *ACS Appl. Mater. Interfaces* **12**, 23207–23216 (2020).

20. X. Liao, W. Song, X. Zhang, C. Yan, T. Li, H. Ren, C. Liu, Y. Wang, Y. Zheng, A bioinspired analogous nerve towards artificial intelligence. *Nat. Commun.* **11**, 268 (2020).

21. M. Nayeri, M. T. Aronson, D. Bernin, B. F. Chmelka, A. Martinelli, Surface effects on the structure and mobility of the ionic liquid C_6C_1ImTFSI in silica gels. *Soft Matter* **10**, 5618–5627 (2014).

22. V. Amoli, J. S. Kim, E. Jee, Y. S. Chung, S. Y. Kim, J. Koo, H. Choi, Y. Kim, D. H. Kim, A bioinspired hydrogen bond-triggered ultrasensitive ionic mechanoreceptor skin. *Nat. Commun.* **10**, 4019 (2019).

23. S. Sharma, A. Chhetry, S. Zhang, H. Yoon, C. Park, H. Kim, M. Sharifuzzaman, X. Hui, J. Y. Park, Hydrogen-bond-triggered hybrid nanofibrous membrane-based wearable pressure sensor with ultrahigh sensitivity over a broad pressure range. *ACS Nano* **15**, 4380–4393 (2021).

24. T. Ji, C. Ma, L. Brisbin, Y. Dong, J. Zhu, Effect of interface on the mechanical behavior of polybutadiene-silica composites: An experimental and simulation study. *J. Appl. Polym. Sci.* **135**, 46089 (2018).

25. J. Kiefer, J. Fries, A. Leipertz, Experimental vibrational study of imidazolium-based ionic liquids: Raman and infrared spectra of 1-ethyl-3methylimidazolium bis(trifluoromethylsulfonyl) imide and 1-ethyl-3-methylimidazolium ethylsulfate. *Appl. Spectrosc.* **61**, 1306–1311 (2007).

26. J. Wu, Understanding the electric double-layer structure, capacitance, and charging dynamics. *Chem. Rev.* **122**, 10821–10859 (2022).

27. M. B. Singh, R. Kant, Debye-Falkenhagen dynamics of electric double layer in presence of electrode heterogeneities. *J. Electroanal. Chem.* **704**, 197–207 (2013).

28. E. K. Boahen, B. Pan, H. Kweon, J. S. Kim, H. Choi, Z. Kong, D. J. Kim, J. Zhu, W. B. Ying, K. J. Lee, D. H. Kim, Ultrafast, autonomous self-healable iontronic skin exhibiting piezo-ionic dynamics. *Nat. Commun.* **13**, 7699 (2022).

29. S. K. Lee, Y. W. Cho, J. S. Lee, Y. R. Jung, S. H. Oh, J. Y. Sun, S. Kim, Y. C. Joo. Nanofiber channel organic electrochemical transistors for low-power neuromorphic computing and wide-bandwidth sensing platforms. *Adv. Sci.* **8**, 2001544 (2021).

30. W. Xu, S.-Y. Min, H. Hwang, T.-W. Lee, Organic core-sheath nanowire artificial synapses with femtojoule energy consumption. *Sci. Adv.* **2**, e1501326 (2016.

31. G.-T. Go, Y. Lee, D.-G. Seo, M. Pei, W. Lee, H. Yang, T.-W. Lee, Achieving microstructure-controlled synaptic plasticity and long-term retention in ion-gel-gated organic synaptic transistors. *Adv. Intell. Syst.* **2**, 2000012 (2020).

32. A. Laiho, L. Herlogsson, R. Forchheimer, X. Crispin, M. Berggren, Controlling the dimensionality of charge transport in organic thin-film transistors. *Proc. Natl. Acad. Sci. U.S.A.* **108**, 15069–15073 (2011).

33. A. Giovannitti, D.-T. Sbircea, S. Inal, C. B. Nielsen, E. Bandiello, D. A. Hanifi, M. Sessolo, G. G. Malliaras, I. McCulloch, J. Rivnay, Controlling the mode of operation of organic transistors through side-chain engineering. *Proc. Natl. Acad. Sci. U.S.A.* **113**, 12017–12022 (2016).

34. D. Rawlings, E. M. Thomas, R. A. Segalman, M. L. Chabinyc, Controlling the doping mechanism in poly (3-hexylthiophene) thin-film transistors with polymeric ionic liquid dielectrics. *Chem. Mater.* **31**, 8820–8829 (2019).

35. W. L. Jorgensen, D. S. Maxwell, J. Tirado-Rives, Development and testing of the OPLS all-atom force field on conformational energetics and properties of organic liquids. *J. Am. Chem. Soc.* **118**, 11225–11236 (1996).

36. W. D. Cornell, P. Cieplak, C. I. Bayly, I. R. Gould, K. M. Merz, D. M. Ferguson, D. C. Spellmeyer, T. Fox, J. W. Caldwell, P. A. Kollman, A second generation force field for the simulation of proteins, nucleic acids, and organic molecules. *J. Am. Chem. Soc.* **117**, 5179–5197 (1995).

37. S. Plimpton, Fast parallel algorithms for short-range molecular-dynamics. *J. Comput. Phys.* **117**, 1–19 (1995).

38. S. A. Mian, L. C. Saha, J. Jang, L. Wang, X. Gao, S. Nagase, Density functional theory study of catechol adhesion on silica surfaces. *J. Phys. Chem. C* **114**, 20793–20800 (2010).

39. W. C. Swope, H. C. Andersen, P. H. Berens,K. R. Wilson, A computer simulation method for the calculation of equilibrium constants for the formation of physical clusters of molecules: Application to small water clusters. *J. Chem. Phys.* **76**, 637–649(1982).

40. R. W. Hockney, J. W. Eastwood, *Computer Simulations Using Particles* (CRC Press, 1988).

41. G. Kresse, J. Furthmüller, Efficient iterative schemes for ab initio total-energy calculations using a plane-wave basis set. *Phys. Rev. B* **54**, 11169–11186 (1996).

42. N. Troullier, J. L. Martins, Efficient pseudopotentials for plane-wave calculations. II. Operators for fast iterative diagonalization. *Phys. Rev. B* **43**, 8861–8869 (1991).

43. H. J. Monkhorst, J. D. Pack, Special points for Brillouin zone integrations. *J. Mater. Chem. A* **13**, 5188–5192 (1976).

44. C. J. Brinker, Hydrolysis and condensation of silicates: Effects on structure. *J. Non Cryst. Solids* **100**, 31–50 (1988).

45. C. H. Chan, H.-W. Kammer, Polymer electrolytes—relaxation and transport properties. *Ionics* **21**, 927–934 (2015).

46. Y. Zang, H. Shen, D. Huang, C.-A. Di, D. Zhu, A dual-organic-transistor-based tactile-perception system with signal-processing functionality. *Adv. Mater.* **29**, 1606088 (2017).

47. D.-G. Seo, Y. Lee, G.-T. Go, M. Pei, S. Jung, Y. H. Jeong, W. Lee, H.-L. Park, S.-W. Kim, H. Yang, C. Yang, T.-W. Lee, Versatile neuromorphic electronics by modulating synaptic decay of single organic synaptic transistor: From artificial neural networks to neuro-prosthetics. *Nano Energy* **65**, 104035 (2019.

48. Y. Liu, W. Yang, Y. Yan, X. Wu, X. Wang, Y. Zhou, Y. Hu, H. Chen, T. Guo, Self-powered high-sensitivity sensory memory actuated by triboelectric sensory receptor for real-time neuromorphic computing. *Nano Energy* **75**, 104930 (2020).

49. R. Yu, Y. Yan, E. Li, X. Wu, X. Zhang, J. Chen, Y. Hu, H. Chen, T. Guo, Bi-mode electrolyte-gated synaptic transistor via additional ion doping and its application to artificial nociceptors. *Mater. Horiz.* **8**, 2797–2807 (2021).

50. Q. Chen, X. Zhang, Y. Liu, Y. Yan, R. Yu, X. Wang, Z. Lin, H. Zeng, L. Liu, H. Chen, T. Guo, Neuromorphic display system for intelligent display. *Nano Energy* **94**, 106931 (2022).

51. C. Jiang, J. Liu, L. Yang, J. Gong, H. Wei, W. Xu, A flexible artificial sensory nerve enabled by nanoparticle-assembled synaptic devices for neuromorphic tactile recognition. *Adv. Sci.* **9**, 2106124 (2022

52. H. Shim, S. Jang, J. G. Jang, Z. Rao, J.-I. Hong, K. Sim, C. Yu, Fully rubbery synaptic transistors made out of all-organic materials for elastic neurological electronic skin. *Nano Res.* **15**, 758–764 (2022).
